# Bacteria elevate extracellular adenosine to exploit host signaling for blood-brain barrier disruption

**DOI:** 10.1080/21505594.2020.1797352

**Published:** 2020-08-10

**Authors:** Zunquan Zhao, Xueyi Shang, Ying Chen, Yuling Zheng, Wenhua Huang, Hua Jiang, Qingyu Lv, Decong Kong, Yongqiang Jiang, Peng Liu

**Affiliations:** a State Key Laboratory of Pathogens and Biosecurity, Institute of Microbiology and Epidemiology, Beijing, China; b Department of Critical Care Medicine, The Fifth Medical Center of Chinese PLA General Hospital, Beijing, China; c School of Food and Chemical Engineering, Beijing Technology and Business University, Beijing, China

**Keywords:** Meningitis, blood-brain barrier, adenosine, *Streptococcus suis*, *Streptococcus agalactiae*, brain microvascular endothelial cell, central nervous system

## Abstract

Bacterial meningitis remains a substantial cause of mortality worldwide and survivors may have severe lifelong disability. Although we know that meningeal bacterial pathogens must cross blood-central nervous system (CNS) barriers, the mechanisms which facilitate the virulence of these pathogens are poorly understood. Here, we show that adenosine from a surface enzyme (Ssads) of *Streptococcus suis* facilitates this pathogen’s entry into mouse brains. Monolayer translocation assays (from the human cerebrovascular endothelium) and experiments using diverse inhibitors and agonists together demonstrate that activation of the A1 adenosine receptor signaling cascade in hosts, as well as attendant cytoskeleton remodeling, promote *S. suis* penetration across blood-CNS barriers. Importantly, our additional findings showing that Ssads orthologs from other bacterial species also promote their translocation across barriers suggest that exploitation of A1 AR signaling may be a general mechanism of bacterial virulence.

## Introduction

Despite advances in antibiotic therapies and vaccinations, the meningitis burden remains high and progress lags substantially behind that of other vaccine-preventable diseases [1,2]. Globally in 2016, incident cases of meningitis were 2.82 million (2.46–3.31), causing 318,400 (265,218–408,705) deaths and 21.87 million (18.20–28.28) disability adjusted life years [2]. Bacterial meningitis is considered the most severe form of this disease and can rapidly become fatal or lead to severe disability in those who survive [2,3]. Bacterial meningitis is predominantly caused by extracellular pathogens; in infants, the most common causative agents are *Escherichia coli* K1 and *Streptococcus agalactiae* (Group B *Streptococcus*, GBS); in children and adults, *Neisseria meningitidis, Haemophilus influenzae* type b (Hib), and *Streptococcus pneumoniae* are most common [4,5]. Most cases of bacterial meningitis occur following bacteremia [1,4,6], and translocation of circulating bacteria across blood-CNS barriers is integral to the pathogenesis of bacterial meningitis.

It is generally accepted that bacteria can cross blood-CNS barriers through three general routes: transcellular, paracellular, and/or Trojan-horse mechanisms [7]. Notably, these routes are not mutually exclusive [5], as shown by evidence from studies of *Neisseria meningitidis* [8,9], GBS [10–12], and *Escherichia. coli* K1 [13,14]. Given that blood-CNS barriers including the blood-brain barrier (BBB) have highly restricted permeability [15], extensive research has examined technologies for targeting smart drug delivery across such barriers, and this work has identified apparent functional contributions from extracellular adenosine in promoting BBB permeability. Specifically, it has been shown that extracellular adenosine can activate the A1 and A2A adenosine receptor proteins (ARs) which are present on the surface of brain endothelial cells [15–20].

Our previous work demonstrated that meningeal bacteria express an enzyme on their surfaces which hydrolyzes AMP to produce adenosine; specifically, we identified the Ssads 5ʹ-nucleotidase from *Streptococcus suis* serotype 2 (*S. suis* 2); for context, *S. suis* 2 is known to cause meningitis and streptococcal toxic shock-like syndrome in humans [21,22], and is a major cause of meningitis in several regions of Asia [23]. Moreover, other studies have reported that surface-exposed adenosine synthases of other meningeal bacteria like *Staphylococcus aureus* and GBS functionally contribute to adenosine production in *in vivo* models of bacterial infection [24–26]. Despite these demonstrations, any functional contributions of these surface-localized adenosine metabolism enzymes on the ability of pathogenic bacteria to promote their translocation across blood-CNS barriers remain unknown.

Here, we demonstrate *in vivo* that *ssads* gene deficiency impedes *S. suis* entry into mouse brains. Using *in vitro* translocation assays with monolayers derived from human brain microvascular endothelial cells we found that Ssads-mediated adenosine production facilitates *S. suis* translocation. We also show – via translocation assays and a variety of *in vivo* chemical complementation experiments that activation or blockade of the A1 adenosine receptor signaling cascade – that engagement between bacterial adenosine production and host A1 AR signal transduction promotes the ability of *S. suis* to enter the CNS. Specifically, we used monolayers prepared from CRISPR-edited human brain endothelial cells and newly generated crisper knockout mice to show that Ssads enzymatic activity and A1 AR signaling work in concert to promote *S. suis* penetration across blood-CNS barriers. Excitingly, we extended these findings by conducting a series of phylogenetic, enzymology, and both *in vitro* and *in vivo* studies which clearly demonstrate that adenosine production by surface-localized Ssads ortholog enzymes from meningeal bacterial species also promote their translocation across the barriers.

## Materials and methods

### Bacteria and human cells

*S. suis* strain 05ZYH33 and the Ssads-deficient mutant strain Δ*ssads* have been described previously [23]. The GBS strain used in this study was a highly virulent clinical strain that we recently isolated from the CSF of a fatal meningitis case, and was identified as a GBS serotype III ST-17 strain by whole-genome sequencing. The *L. monocytogenes* strain used was 10403s, the *S. aureus* strain used was USA300, the *S. epidermidis* and *S. pneumoniae* strains used were clinical isolates. Briefly, *S. suis* and GBS were grown in THY and THB medium (Bacto), respectively, at 37°C under an atmosphere containing 5% CO_2_ without shaking. The immortalized human brain microvascular endothelial cell line (HCMEC/D3) was obtained under license from INSERM, France, and cultured in EBM-2 (Lonza) or ECM (Sciencell) endothelial cell media containing 5% FBS (fetal bovine serum; Gibco), and supplemented as previously described [27].

### Chemicals and reagents

APCP (a known 5ʹ-nucleotidase inhibitor), DPCPX (a selective A1 AR antagonist), ZM 241385 (a potent selective A2A AR antagonist), CCPA (a selective A1 AR agonist), AMP, and adenosine were purchased from Sigma-Aldrich. Rp-cAMPS (a competitive antagonist of cAMP-induced PKA activation) and PSB 603 (a potent selective A2B AR antagonist) were purchased from TOCRIS. NECA (a nonselective AR agonist) was purchased from MedChemExpress.

### Mouse model of hematogenous meningitis

The challenge protocol for *S. suis* infection was adapted from a murine model of *S. suis* meningitis using an intraperitoneal infection route [28,29]. Some modifications were also made to prolong the duration of severe *S. suis* bacteremia and to reduce acute death during the early stages of infection. Briefly, six-week-old female C57BL/6 mice (Vital River) or C57BL/6 A1 AR-KO mice were injected intraperitoneally (i.p.) with 1 mL of the bacterial suspensions (5 × 10^6^ colony-forming units (CFU)/mL) or vehicle alone (sterile THY media). Mice were euthanized and samples of venous blood and brain were collected aseptically 72 h post-infection. Bacterial counts in blood and tissue homogenates were determined by plating serial dilutions on THB agar plates. Brain bacterial counts were corrected for blood contamination using the blood concentration and a conservative estimate of the mouse cerebral blood volume (2.5 mL per 100 g tissue) [30–33]. For the comparison between i.p. and i.v. infection route, some six-week-old female C57BL/6 mice were injected via the tail vein with 200 μL of bacterial suspensions (5 × 10^8^ CFU/mL) in PBS.

In some experiments, brain samples or brain hemispheres were collected for histopathologic analysis. For GBS infection, a well-defined and widely used mouse model of hematogenous GBS meningitis was used as described previously [10,30–33]; details are provided in Supplementary Methods. Bacterial CFUs in venous blood were examined at 16 h and 72 h post-infection and brain samples were collected aseptically at 72 h post-infection. CCPA (0.37 mg/kg BW) or vehicle was administered intravenously (i.v.) at 2 h post-infection. DPCPX (1 mg/kg BW) or vehicle was injected i.p. concomitantly with bacterial cells as described in previous studies [34,35]. APCP (20 mg/kg BW) or vehicle was injected i.p. 2 h prior to infection as described in previous studies [36,37]. The A1 AR-KO mice were obtained by using the CRISPR/Cas9 technique from Cyagen Biosciences, using the targeting strategy described at https://www.cyagen.com/cn/zh-cn/sperm-bank/11539.

### Translocation of S. suis across microvascular endothelial monolayers

Briefly, HCMEC/D3 monolayers were cultured on the apical side of collagen-coated 3.0-μm pore Millicell inserts (Merck Millipore) in 24-well plates (Corning) [18]. The multiplicity of infection (MOI) used was 100 [8]. After 1 h of infection, medium from the lower chamber was collected and sampled to quantitate the number of viable bacteria. For Lucifer yellow (LY) permeability, LY (50 μM) was added to upper chamber. The LY fluorescence of the media in lower chamber was measured by MD SpectraMax i3 system (Molecular Devices) at absorption/emission of 428/536 nm. TEER values were measured using the Millicell® ERS-2 Electrical Resistance System (Merck Millipore). Details are provided in the Supplementary Methods.

### Determination of the intracellular cAMP concentration

The intracellular cAMP concentration was assayed using a cAMP parameter assay kit (R&D Systems). Details are provided in the Supplementary Methods.

### Knockout using CRISPR/Cas9 genome editing

*Adora1* knockout (KO) cells were generated from HCMEC/D3 cells using CRISPR/Cas9 genome editing as described previously [38,39]. Details are provided in the Supplementary Methods.

### Isolation of primary murine primary brain microvascular endothelial cells

Primary murine brain microvascular endothelial cells (pMBMECs) were isolated and purified using a well-established and previously described method [30]. Details are provided in the Supplementary Methods.

### Western blotting

Western blotting was performed using routine methods. Primary antibodies against the following proteins were used: mouse anti-GAPDH (1:5,000; Invitrogen MA5-15738), and rabbit anti-A1 AR (1:200; Alomone AAR-006). Secondary antibodies were used: IRDye 800CW-conjugated goat anti-rabbit IgG antibody (1:5,000; Li-Cor 926–32211), or IRDye® 680RD-conjugated goat anti-mouse IgG antibody (1:5,000; Li-Cor 926–68070). Protein bands were visualized using an Odyssey Infrared Imaging System (Li-Cor Biosciences). Details are provided in the Supplementary Methods.

### Immunocytochemistry

The pMBMEC cells were grown on fibronectin-coated Millicell EZ SLIDEs 4-well glass slides (Merck Millipore). The multiplicity of infection (MOI) used was 10. After 5 h of infection, immunofluorescence staining was performed using routine methods. Details are provided in the Supplementary Methods.

### 5ʹ-nucleotidase activity assay

The 5ʹ-nucleotidase activity of Ssads and apparently homologous enzymes from other meningeal species (GBS, *S. aureus*, or *S. epidermidis, L. monocytogenes*, and *S. pneumoniae*) was measured by detection of inorganic phosphate released from AMP hydrolysis using a QuantiChrom Phosphate Assay Kit DIPI-500 (Bioassay systems). Details are provided in the Supplementary Methods.

### Statistics

Statistical analysis was performed using Prism 5.0 (GraphPad Software). For bacterial count data in animal experiments, representative data are from experiments which were replicated biologically at least 3 times with similar results; these non-normally distributed count data values are presented as the median ± IQR (interquartile range), and Mann-Whitney *U* tests were used to determine the significance of count differences. For other experiments, the mean with the SEM (standard error of the mean) of at least three independent experiments is provided. To test for significance of differences, a paired two-tailed Student’s *t* test or a two-way analysis of variance (ANOVA) with Bonferroni multiple comparison test was performed. Partial data were not corrected for multiple testing, because the analyses were not ad hoc but at each stage address a series of specific hypotheses, each of which is based on a priori knowledge of underlying mechanisms [40–42]. A *P* value less than 0.05 was considered statistically significant.

### Ethics statement

All animals were cared for in accordance with the principles in the Guide for the Care and Use of Laboratory Animals of the National Institutes of Health. The protocol of animal study was approved by the IACUC of the Academy of Military Medical Sciences (IACUC of AMMS-13-2016-013). For all experiments, every effort was made to minimize the suffering of the animals.

## Results

### Ssads gene deficiency impedes S. suis entry into mouse brains

To investigate the role of Ssads-mediated adenosine production in meningitis pathogenesis, we compared the relative virulence of *S. suis* wild-type (WT) strain 05ZYH33 and the isogenic *ssads*-deficient mutant Δ*ssads* in a murine model of *S. suis* meningitis via intraperitoneal infection route. *S. suis* colony-forming units (CFU) counts in the blood rose to a high and broad peak (about 10^8^ CFU/mL) around 5 h (hours) post-infection, and then decreased gradually to low levels (about 10^5^ CFU/mL) (Figure S1(a)). No significant difference was observed between bacterial loads in the blood of *S. suis* WT- and Δ*ssads*-infected mice during the early stages of infection (1 h to 6 h post-infection) (Figure S1(a)). Compared to the intraperitoneal infection route, those mice injected i.v. with 1 × 10^8^ CFU *S. suis* WT strain or *S. suis* Δ*ssads* showed relatively low-grade bacteremia during the first 24 hours post infection (Figure S1(b)). Therefore, the intraperitoneal challenge method was selected to build murine model of *S. suis* meningitis. Additionally, we confirmed that the growth rate of the *S. suis* Δ*ssads* is comparable to that of the *S. suis* WT strain (Figure S2(a) to S2(b)) in THY medium.

**Figure 1.**
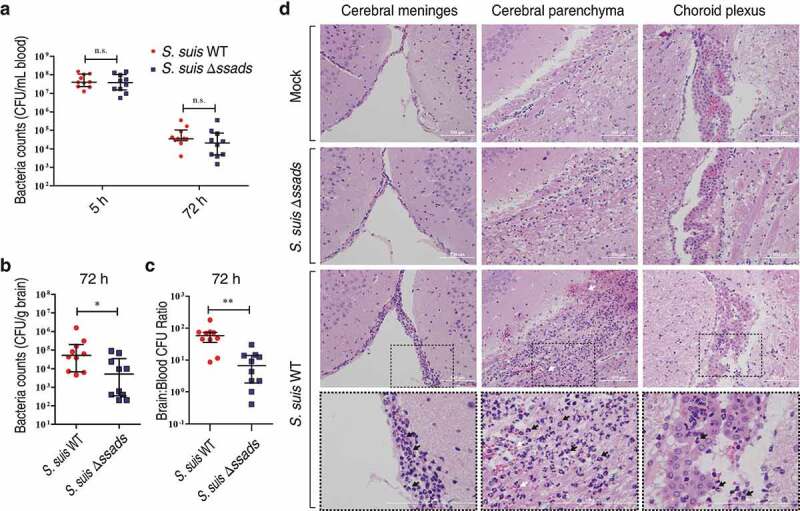
***Ssads* gene deficiency impedes *S. suis* entry into mouse brains**. (a–c) Six-week-old female C57BL/6 mice were infected i.p. with approximately 5 × 10^6^ CFU of the *S. suis* WT strain 05ZYH33 (*n* = 10), the *ssads*-deficient mutant Δ*ssads* (*n* = 10), or sterile THY media (*n* = 3). Bacterial counts in the blood (CFU/mL blood) at 5 h and 72 h post-infection (a) and brain (CFU/g tissue) at 72 h post-infection (b) were determined. (c) After correction for blood contamination using CFU in blood and a conservative estimate of the mouse cerebral blood volume (2.5 mL per 100 g tissue), the ratio of brain:blood CFU at 72 h post-infection was determined. Horizontal lines and error bars denote the median and IQR, respectively. *P < 0.05, **P < 0.01, Mann-Whitney *U* test. (d) Histopathology of representative brain tissues from mice injected with THY media (Mock), infected with *S. suis* WT strain or infected with the Δ*ssads* mutant. Dotted box regions are magnified to better indicate hemorrhage (white arrows) and polymorphonuclear leukocyte infiltration (black arrows). Scale bar indicates 100 µm.

In subsequent experiments, we quantified bacterial loads in blood at 5 h post-infection to ensure consistent measurement of bacteremia and found that bacterial loads in the blood of *S. suis* WT- and Δ*ssads*-infected mice at 5 h post-infection were nearly identical (Figure 1(a)). However, quantification of bacterial load from homogenized brains of animals sacrificed at 72 h post-infection revealed that the brains of the mice infected with the Δ*ssads* mutant had significantly lower bacterial loads than the brains of mice infected with the *S. suis* WT strain, even when carefully deducting signal from blood-residing bacterial cells (Figure 1(b)). In fact, bacterial loads in the blood at 72 h were at relatively low levels (Figure 1(a)), based on a conservative estimate of the mouse cerebral blood volume (2.5 mL per 100 g tissue) [30–33], and would have interfered little with corresponding bacterial counts in the brain (Figure 1(b)). The ratio of brain:blood CFU in mice infected with the Δssads mutant was also significantly lower than that of mice infected with the *S. suis* WT strain (Figure 1(c)). Moreover, histological analysis showed significantly increased meningeal thickness, hemorrhaging, and inflammatory cell infiltration in the animals infected with the WT strain; mice infected with the Δ*ssads* strain showed normal brain morphology with few signs of injury or inflammation (Figure 1(d)). Together, these results indicate that the *ssads* gene enhances the infectivity of the meningeal bacteria *S. suis* 2.

### Ssads-mediated adenosine production facilitates S. suis translocation across HCMEC/D3 monolayers

**Figure 2.**
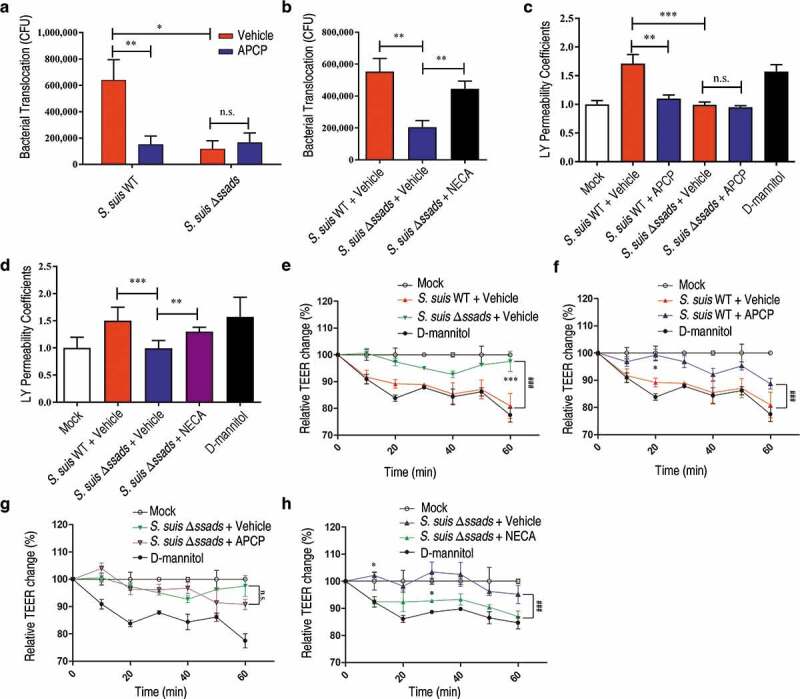
**The 5ʹ-nucleotidase activity of Ssads contributes to *S. suis* translocation across the monolayers derived from human brain microvascular endothelial cells**. After HCMEC/D3 cells formed confluent and tight monolayers on transwell filters, *S. suis* strains were added to the apical surfaces of monolayers at an MOI of 100. (a) In the presence or absence of APCP (500 µM), a known 5ʹ-nucleotidase inhibitor, *S. suis* cells in the lateral chamber after 1 h were quantitated. (b) The effect of the adenosine analog NECA (1 µM) on bacterial translocation across HCMEC/D3 monolayers at 1 h post-treatment was determined. (c–d) The permeability coefficient to Lucifer yellow (LY) of HCMEC/D3 monolayers was measured 1 h post-infection with *S. suis* in the presence or absence of APCP (500 µM; c) or NECA (1 µM; d). Data are expressed as means and SEM. *P < 0.05, **P < 0.01, ***P < 0.001, 2-tailed Student’s *t* test. (e–h) The TEER in HCMEC/D3 monolayers infected with *S. suis* in the presence or absence of APCP (500 µM) (e–g) or NECA (1 µM) (h) was measured. D-mannitol (10 µM) was used as a positive control as it disrupts cell-cell junctions. Data are expressed as means and SEM. ^###^*P* < 0.001 analyzed by two-way ANOVA, and the following significant differences (*P < 0.05, ***P < 0.001 by Bonferroni multiple comparison test) between the corresponding groups at the indicated time points are displayed.

As *ssads* gene deficiency impeded *S. suis* entry into the brains of mice, we next examined the effects of Ssads-mediated adenosine production on the translocation of *S. suis* cells using an *in vitro* experimental system. Using previously described methods, we employed a well-characterized immortalized HCMEC/D3 cell line to generate a transwell model of the human brain endothelial cell monolayer [8]. To study the impact of surface adenosine production on the translocation ability of *S. suis* across endothelial monolayers, WT and Δ*ssads S. suis* cells were added to the apical surfaces of the HCMEC/D3 monolayers at an MOI (multiplicity of infection) of 100, a level selected to appropriately approximate known bacteremia levels at 5 h post-infection in animal experiments (Figure S1(a) and S2(c)). No significant difference of growth rate was observed between WT and Δ*ssads S. suis* cells cultured in upper chamber medium (Figure S2(c)). *ssads* deficiency significantly reduced the *S. suis* counts in the basolateral chamber after 1 h (Figure 2(a)), but had no significant effect on bacterial adhesion to or invasion of HCMEC/D3 cells (Figure S3). Further, inhibition of Ssads 5ʹ-nucleotidase activity using adenosine 5ʹ-(α,β-methylene)diphosphate (APCP) – a known 5ʹ-nucleotidase inhibitor – also significantly reduced the translocation of the *S. suis* WT strain across the monolayer, supporting that the catalytic activity of Ssads directly contributes to the observed reduction in translocation. Additionally, chemical complementation assays showed that the reduced translocation phenotype of the Δ*ssads* mutant across the monolayers was rescued by the exogenous application of the adenosine analog 5ʹ-(N-Ethylcarboxamido)adenosine (NECA) but not by the control vehicle-alone treatment (Figure 2(b)). The addition of NECA did not significantly affect the translocation of *S. suis* WT strain across the monolayer (Figure S4(a)).

To examine changes in endothelial monolayer permeability, we used a previously described approach to conduct passive paracellular diffusion assays based on the fluorescent marker molecule Lucifer yellow (LY) [8]. Addition of the *S. suis* WT strain to monolayers increased their permeability to LY, whereas the Δ*ssads* mutant had no significant effect on permeability (Figure 2(c)). The increased permeability to LY induced by *S. suis* WT strain was dependent on the enzymatic activity of Ssads, as the effect was eliminated by addition of APCP (Figure 2(c)), and a functional role for local adenosine was supported by our finding that the exogenous application of NECA to the samples with the Δ*ssads* mutant (Figure 2(d)) but not *S. suis* WT (Figure S4(b)) significantly increased permeability.

The transendothelial cell electrical resistance (TEER) was measured to further evaluate the barrier permeability of the HCMEC/D3 monolayers [15]. Decreased TEER in endothelial cell monolayers was observed after addition of both strains, but a significantly more pronounced decrease was measured for *S. suis* WT strain compared to the Δ*ssads* mutant (Figure 2(e)). The APCP treatment neutralized the TEER descent caused by the *S. suis* WT infection (Figure 2(f)) but not the Δ*ssads* mutant infection (Figure 2(g)). We also observed that the exogenous application of NECA assisted Δ*ssads* mutant in decreasing TEER (Figure 2(h)), but did not affect the TEER alteration induced by the *S. suis* WT infection (Figure S4(c)). Collectively, these results demonstrate that Ssads-mediated adenosine production can increase the permeability of endothelial monolayers and suggest that such production facilitates *S. suis* translocation across these monolayers.

### Ssads-mediated adenosine production results in rearrangement of the actin cytoskeleton and endothelial junctions

**Figure 3.**
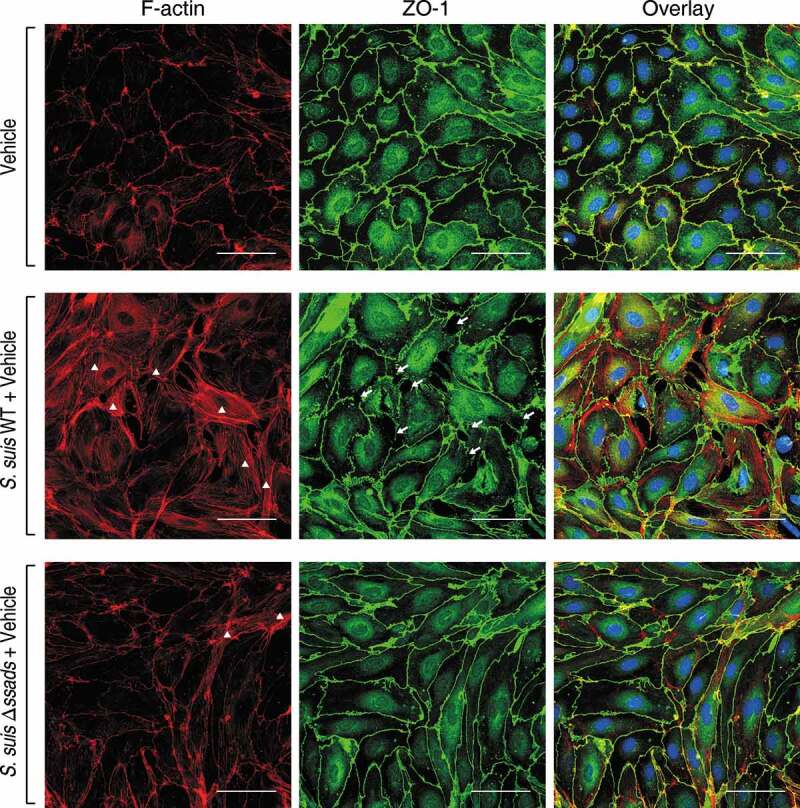
**Ssads-mediated adenosine production induces cytoskeletal reorganization and JPs redistribution in *S. suis*-infected pMBMECs**. Primary murine brain microvascular endothelial cells (pMBMECs) were isolated, purified, and cultured from six-week-old female C57BL/6 mice. (a) Immunofluorescence assay for F-actin (red) and ZO-1 (green) in pMBMEC cells was performed 5 h post-infection with *S. suis* strains WT or Δ*ssads*. Nuclei were counter stained with DAPI (blue). Robust short stress fiber formation (white triangle) was visualized using phalloidin staining of F-actin. White arrows indicate paracellular gaps. Scale bar indicates 50 µm.

Dynamic interactions between the actin cytoskeleton and junctional proteins (JPs) are required for maintenance of cell shape and endothelial barrier integrity [16], and it is well known that robust actin polymerization and stress fiber formation in endothelial cells alters cell morphology and redistribution/disassembly of JPs, thereby increasing barrier permeability [43]. We therefore investigated the potential function of Ssads in intracellular actin dynamics that may underlie changes in the permeability of endothelial monolayers after *S. suis* infection. Compared with immortalized endothelial cell line [44], primary endothelial cells have advantages to study subtle barrier properties such as rearrangement of actin cytoskeleton and tight junctions [30,43,45,46]. We isolated and purified primary murine brain microvascular endothelial cells (pMBMECs) from C57BL/6 mice and infected them *ex vivo* with the WT and Δ*ssads S. suis* strains. The staining (green) of Zonula Occludens 1 protein (ZO-1), a kind of tight junction protein, indicated clear and vivid outlines of pMBMECs (Figure 3). As the actin cytoskeleton morphology of those uninfected pMBMECs displayed, intracellular red staining (phalloidin) was distributed throughout the cytoplasm as faint and diffuse state, except that the gathered red only approached the outline of the cell (Figure 3). It is known that the cytoplasmic actin that is normally short filaments and diffuse monomers (G-actin) would be polymerized into F-actin, which can be clearly stained by phalloidin, and even linear stress fibers (polymerized F-actin) across the cell interior following exposure to specific stressors such as hypoxia, free radicals [43]. Phalloidin staining for F-actin revealed robust stress fiber formation in the cytoplasm at 5 h post-infection with the *S. suis* WT strain (Figure 3, white triangles). In contrast, the Δ*ssads*-infected pMBMECs exhibited fewer stress fibers in the cytoplasm, and had a cortical distribution of the cytoskeletal F-actin filaments similar to control cells treated with vehicle alone (Figure 3). Moreover, weakness of tight junctions, as shown by increased discontinuous staining on the lateral membrane and appearance of paracellular gaps, was observed by immunostaining for ZO-1 5 h after the infection with *S. suis* WT strain (Figure 3, white arrows).

### Translocation of S. suis across blood-CNS barriers involves adenosine receptor signaling

**Figure 4.**
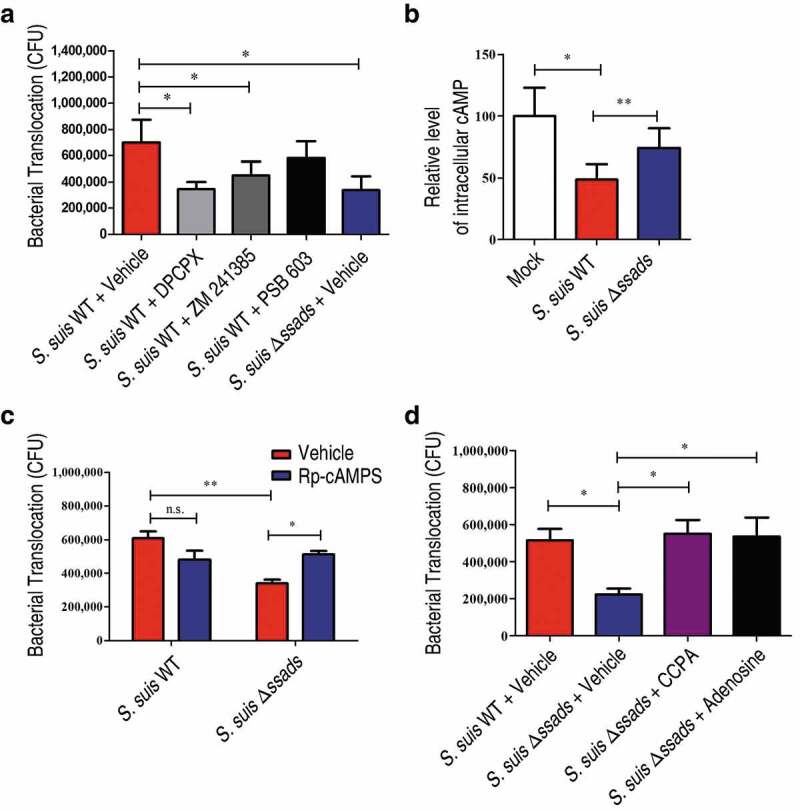
**Ssads-mediated AR signaling facilitates *S. suis* translocation across the BBB *in vitro***. *S. suis* strains were added to confluent HCMEC/D3 monolayers at an MOI of 100. (a) The effects of selective AR antagonists DPCPX (100 nM; specific for A1 AR), ZM 241385 (1 µM; specific for A2A AR), and PSB 603 (1 µM; specific for A2B AR) on bacterial translocation across HCMEC/D3 monolayers at 1 h post-infection were determined. (b) The cellular cAMP content of HCMEC/D3 cells was measured 1 h post-infection with *S. suis* cells. (c) The effects of Rp-cAMPS (200 µM) – a competitive antagonist of cAMP-induced activation of PKA – on bacterial translocation across HCMEC/D3 monolayers at 1 h post-infection were determined. (d) The effects of the selective A1 AR agonist CCPA (1 µM) and adenosine (10 µM) on bacterial translocation across HCMEC/D3 monolayers at 1 h post-infection were determined. Data are expressed as means ± SEM. *P < 0.05, **P < 0.01, ***P < 0.001, two-tailed Student’s *t* test.

Humans have four different AR proteins: A1, A2A, A2B, and A3, and HCMEC/D3 cells express three of them (A1, A2A, and A2B) [16,19]. We next evaluated the translocation of *S. suis* across endothelial monolayers in the presence of adenosine receptor antagonists, and found that the selective A1 AR antagonist 8-Cyclopentyl-1,3-dipropylxanthine (DPCPX) significantly reduced the translocation of the *S. suis* WT strain across monolayers. Further, treatment with the potent selective A2A AR antagonist ZM 241385 exerted a weak but significant inhibitory effect on the translocation of the *S. suis* WT strain across endothelial monolayers (Figure 4(a)). In contrast, treatment with the potent selective A2B AR antagonist PSB 603 (1 μM; a working concentration for inhibiting the effect of NECA on HCMEC/D3 cells [19]) did not significantly affect translocation of *S. suis* (Figure 4(a)), probably because the local adenosine concentration in transwell model was not high enough to activate the low affinity A2B AR. Thus, it appears that A1 and/or A2A may participate in the observed Ssads-mediated alteration of the translocation capacity of *S. suis* across monolayers.

The four human ARs are all G protein-coupled receptors (GPCRs). A1 initiates a Gα_i_ signaling cascade which ultimately downregulates production of the second messenger cyclic AMP (cAMP), whereas A2A and A2B interact with Gα_s_ proteins known to trigger increases in cellular cAMP [16]. We therefore measured the cellular cAMP content of the HCMEC/D3 cells upon the addition of the two *S. suis* strains. The *S. suis* WT-infected HCMEC/D3 cells exhibited significantly lower levels of cellular cAMP compared to Δ*ssads*-infected cells at 1 h post-treatment (Figure 4(b)). These results, viewed alongside our finding that selective blockage of A1-signaling with the A1 antagonist DPCPX significantly reduces translocation of WT *S. suis*, establish that *S. suis* cells with functional Ssads are able to activate the A1 AR signaling axis in brain microvascular endothelial cells. Moreover, and supporting that cellular processes known to be triggered by the A1 signaling axis contribute to the observed changes in translocation across monolayers, we also observed a significant increase in the translocation of Δ*ssads* mutant cells following treatment with Rp-cAMPS, a competitive antagonist of cAMP that blocks cAMP-induced activation of protein kinase A (PKA) (Figure 4(c)). Importantly, we also found that the translocation phenotype of the Δ*ssads* cells could be rescued upon the addition of the selective A1 AR agonist (CCPA; Figure 4(d)). These results together support the speculation that the local engagement of adenosine with A1 directly functionally promotes *S. suis* capacity to translocate across barriers.

### Ssads enzymatic activity and A1 AR signaling work in concert to promote S. suis penetration across blood-CNS barriers

**Figure 5.**
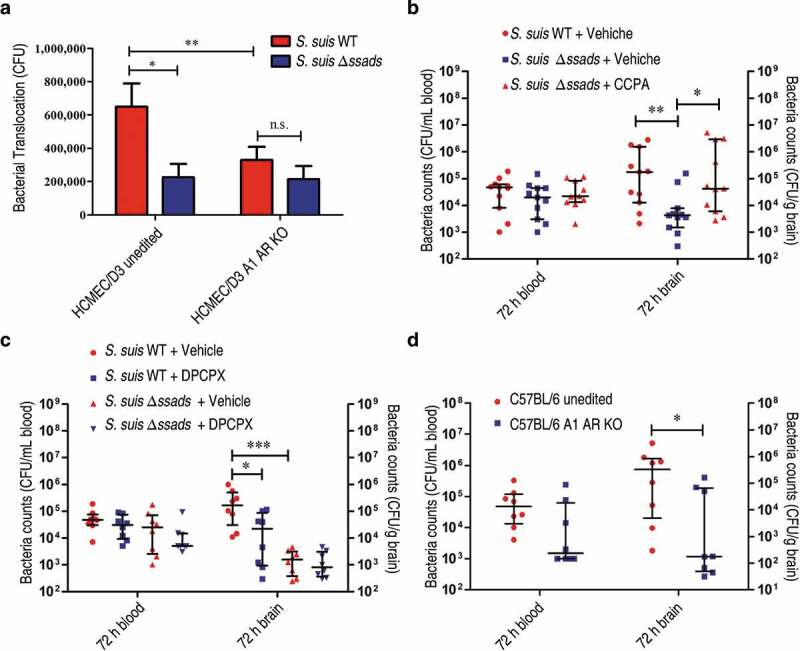
**A1 AR activation promotes *S. suis* BBB penetration**. (a) The translocation of *S. suis* across monolayers of HCMEC/D3 A1 AR-KO cells or HCMEC/D3 unedited cells was measured at 1 h post-infection. Data are expressed as means ± SEM. (b,c) Six-week-old female C57BL/6 mice were infected i.p. with approximately 5 × 10^6^ CFU of *S. suis* strains. Bacterial CFU in the blood and brain were measured from animals sacrificed at 72 h post-infection. Horizontal lines and error bars denote the median and IQR, respectively. (b) The effects of CCPA (0.37 mg/kg BW) i.v. at 2 h post-infection on the bacterial loads in the brain and blood were determined (*n* = 11 mice per group). (c) The effects of administration of DPCPX (1 mg/kg BW) concomitantly with bacterial suspension by the i.p. route on the bacterial loads in the brain and blood were determined (*n* = 8 mice per group). (d) Comparison of bacterial CFU recovered from the blood and recovered from brains of unedited and A1 AR-KO mice 72 h after i.p. challenge with 5 × 10^6^ CFU of *S. suis* WT strain were performed (*n* = 8 mice per group). Horizontal lines and error bars denote the median and IQR, respectively. *P < 0.05, **P < 0.01, ***P < 0.001, Mann-Whitney *U* test.

We knocked out the *ADORA1* locus (encoding A1 AR) in the HCMEC/D3 cell line using CRISPR/Cas9 genomic editing (Figure S5(a) to S5(c)) and then prepared two types of human brain microvascular endothelial monolayers: monolayers from unedited cells and A1 AR-KO monolayers. Translocation assays with these monolayers revealed significantly different translocation rates for the WT versus Δ*ssads* mutant cells across the monolayers prepared from unedited cells, whereas no difference in translocation rates between the strains was observed for the A1 AR-KO monolayers (Figure 5(a)). These results from human cells support that the simultaneous presence of functional Ssads and functional A1 substantially promotes the ability of *S. suis* to translocate across monolayers.

We next explored the *in vivo* role(s) of A1 AR signaling during *S. suis* entry into murine brains. After confirming our previous observation of significantly increased bacterial loads at 72 hours in the brains of the *S. suis* WT-infected C57BL/6 mice compared to the mice infected with Δ*ssads* (Figure 5(b), right), and after again observing no difference in the blood bacterial load between the two experimental groups (Figure 5(b), left), we next conducted chemical complementation experiments using the aforementioned A1-specific agonist compound CCPA. Intravenously administered CCPA (0.37 mg/kg body weight (BW)) completely rescued the reduced brain bacterial load phenotype of the Δ*ssads*-infected mice at 72 h post-infection (Figure 5(b)). Moreover, when the selective A1 antagonist compound DPCPX (1 mg/kg BW) was injected i.p. concomitantly with *S. suis* WT cells, the bacterial loads in brains examined at 72 h were significantly diminished compared to the vehicle controls (Figure 5(c)). We also generated A1 AR-KO mice in collaboration with Cyagen Biosciences (Figure S3(d)) and found that, compared to the brains of *S. suis*-infected unedited mice, the bacterial loads were significantly reduced in the brains of *S. suis*-infected A1 AR-KO mice at 72 h post-infection (Figure 5(d)).

### Adenosine production by other bacterial species facilitated bacterial translocation across the BBB

**Figure 6.**
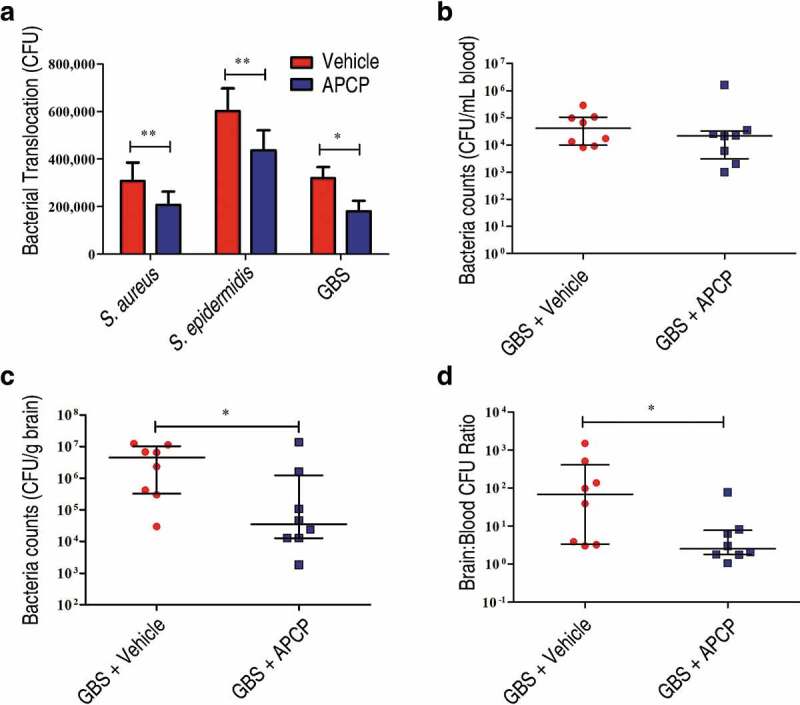
**Adenosine production by other bacterial species is involved in bacterial translocation across the BBB**. (a) The effects of the 5ʹ-nucleotidase inhibitor APCP (500 µM) on the translocation of GBS, *S. aureus*, or *S. epidermidis* across HCMEC/D3 monolayers at 1 h were determined. Data are expressed as means and SEM. *P < 0.05, **P < 0.01, two-tailed Student’s *t* test. (b–d) Six-week-old male CD1 mice were injected via the tail vein with 10^8^ CFU of GBS. The effects of administration of APCP i.p. 2 h prior to challenge on the progression of GBS meningitis were determined. Bacterial burdens in the blood (b) or brain (c), as well as the ratio of brain:blood CFU (d) were determined 72 h post-infection (*n* = 8 mice per group). Brain CFUs were corrected for blood contamination using the blood CFU and a conservative estimate of the mouse cerebral blood volume (2.5 mL per 100 g tissue). Horizontal lines and error bars denote the median and IQR, respectively. *P < 0.05, Mann-Whitney *U* test.

The genomes of several other bacterial species encode putative 5ʹ-nucleotidases (Figure S6; Table S1 to S2). Among Gram-positive pathogenic bacteria, *Listeria monocytogenes*, GBS, *S. aureus*, and *Staphylococcus epidermidis*, all of which can cause meningitis, harbor putative 5ʹ-nucleotidase genes containing LPXTG motifs. Thus, we investigated whether their adenosine synthases could also facilitate bacterial translocation into the CNS. By detecting the release of inorganic phosphate from 50 μM AMP in the presence of divalent metal ions, we confirmed that *L. monocytogenes*, GBS, *S. aureus*, and *S. epidermidis* have adenosine synthase activity (Figure S7). However, *S. pneumoniae* exhibited no enzymatic activity (Figure S7). We next evaluated the translocation ability of GBS, *S. aureus*, and *S. epidermidis* across HCMEC/D3 monolayers in the presence or absence of the aforementioned 5ʹ-nucleotidase inhibitor APCP. All three bacteria could cross the monolayers, and we found that the addition of APCP significantly reduced their translocation ability (Figure 6(a)). In contrast, the addition of APCP did not significantly affect the translocation ability of *S. pneumoniae*, despite that the meningitis pathogen harboring no enzymatic activity could cross both of unedited and A1 AR-KO HCMEC/D3 monolayers (Figure S8).

These results suggest that the catalytic activity of surface-localized Ssads ortholog 5ʹ-nucleotidase contributes to the translocation capacity of many meningeal species. Using a well-defined and widely used mouse model of GBS hematogenous meningitis [10,30–33], we further explored the *in vivo* role of local adenosine synthesis during GBS entry into murine brains. We found that APCP treatment to interrupt adenosine production significantly reduced brain bacterial loads (Figure 6(b–d)) at 72 h post-infection. Thus, our results support that that local adenosine synthesis by meningeal species may alter blood-CNS barrier permeability and facilitate bacterial translocation into the CNS.

## Discussion

**Figure 7.**
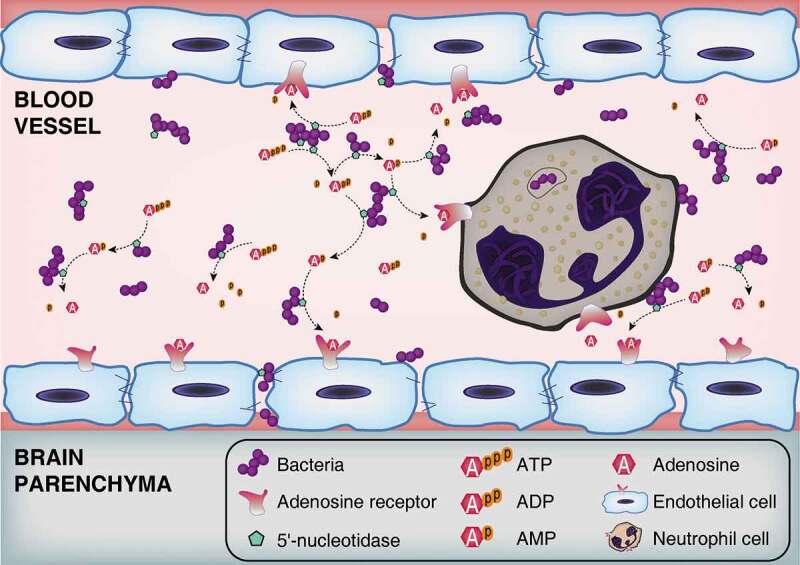
**Schematic model of BBB disruption induced by bacterial 5ʹ-nucleotidase**. Bacteria increase the extracellular concentration of adenosine catalyzed by 5ʹ-nucleotidase. Thereafter, through occupancy of the A2A AR on neutrophil cells, adenosine acts to perturb immune defenses. Abnormally elevated adenosine levels can also destroy the integrity of CNS barriers by activating A1 AR expressed on brain endothelial cells. As bacteria proliferate and adenosine impairs the function of immune and endothelial cells, infected individuals gradually develop bacteremia and meningitis.

Extracellular adenosine has been shown to increase BBB permeability by activating ARs expressed on brain microvascular endothelial cells [16,18]. Our previous work demonstrated that Ssads produces adenosine at the surface of pathogenic bacteria and showed that disruption of this enzyme’s enzymatic function affected the pathogen’s virulence in a sepsis model [23]. Building from this, in the present study we found that genetic disruption of Ssads impeded *S. suis* entry into the brains of mice and show the specific contributions of Ssads and adenosine receptors to the observed differences (Figure 7). It is noteworthy that the effect of Ssads on translocation seems to be independent of bacterial adhesion alteration, and indeed *S. suis* almost could not invade human brain microvascular endothelial cells in spite of their weak adherence, which is consistent with a previous work [47].

Several groups have suggested that *S. suis* enters the CSF from the blood via the choroid plexus (CP). The judgment was primarily based on histologic and immunohistochemical findings of pathologic lesions in CPs from meningitic pigs [48–50] as well as observation of polar *S. suis* invasion and translocation across a CP epithelial monolayer *in vitro* [51]. Aside from the CP, the meninges were also associated with pathologic lesions in these studies [48–50]. In certain regions close to the meninges, the blood-brain barrier (BBB) is relatively vulnerable, as the existence of Virchow-Robin perivascular space between endothelial cells and astrocytes may facilitate bacterial penetration [4,5]. Therefore, the sites of *S. suis* translocation are not necessarily exclusive.

Given that adenosine has a relatively short half-life of less than 10 seconds, extracellular adenosine is short-lived *in vivo* [16,52,53]. The adenosine level of serum is difficult to be detected by HPLC and mass spectrometry. In our previous work and another study [23,24], plasma samples were incubated with bacteria, and subjected to reversed-phase HPLC (RP-HPLC). Ssads-mediated adenosine production was observed in *S. suis* interactions with blood cells [23]. Consistent with previous work [15,16,18], our data revealed that Ssads-mediated AR signaling may induce barrier dysfunction through cytoskeletal remodeling and stress fiber formation in pMBMECs model. Indeed, a recent study reported cytoskeletal alterations in brain endothelial cells as an initiator of BBB rupture after ischemia-reperfusion injury [43]. Our experiments reveal the direct effect of Ssads-mediated AR signaling on the endothelial cell cytoskeleton at an early stage of infection. AR signaling may also affect barrier properties indirectly, as some evidence suggests that adenosine can trigger release of vascular endothelial growth factor (VEGF), a potent inducer of BBB disruption in adults [54], from endothelial cells [55] and immune cells [56,57] through A2A or A2B AR signaling. Thus, the indirect effect of Ssads-mediated adenosine production associated with VEGF release may occur when the local adenosine concentration is high enough for a sustained period during bacteremia.

Among the other bacteria harboring adenosine synthases, GBS, *L. monocytogenes*, Hib, and *E. coli* K1 are leading causes of bacterial meningitis. Our data suggest that GBS expresses a cell-surface adenosine synthase, which is consistent with previous reports [25], and that enzyme activity may facilitate the bacterial entry into CNS. A recent study reported a crucial role of A2A and A2B AR signaling and VEGF production in inducing BBB dysfunction *in vitro* after infection with *H. influenza* type a, but did not mention 5ʹ-nucleotidase [58]. Interestingly, an early study of a subunit vaccine candidate against nontypeable *H. influenza* (NTHi) identified a surface-exposed, highly conserved (both in NTHi and Hib strains) 5ʹ-nucleotidase harboring enzymatic activity [59]. Additionally, a recent study suggested that release of periplasmic 5ʹ-nucleotidase in *E. coli* induced by human β-defensin-2 can also cause accumulation of adenosine [60].

In conclusion, we here demonstrate *in vivo* that deficiency of an adenosine metabolism enzyme (Ssads) expressed at the surface of the meningeal bacteria *S. suis* substantially weakens its capacity to infect mice, and demonstrate that Ssads-mediated adenosine production facilitates *S. suis* translocation across monolayers prepared from human brain microvascular endothelial cells. An extensive assortment of inhibitors and receptor antagonist/agonist experiments showed that activation of the A1 adenosine receptor signaling cascade in hosts – and attendant cytoskeleton remodeling – together support that bacterial adenosine production exploits host A1 AR signal transduction to promote *S. suis* penetration across blood-CNS barriers. We extended these findings by conducting a series of phylogenetic, enzymatic, *in vitro*, and *in vivo* studies which clearly demonstrate that adenosine production of surface-localized Ssads orthologs of other bacterial species also promote translocation across the barriers. In addition to demonstrating functional roles for Ssads from *S. suis* and for host A1 AR signaling in the blood-CNS barrier translocation process, our study strongly suggests that the highly localized production and reception of adenosine may be widely exploited by many pathogenic bacterial species to facilitate host entry and infection.

## Supplementary Material

Supplemental Material

## References

[1] McGill F, Heyderman RS, Panagiotou S, et al. Acute bacterial meningitis in adults. Lancet. 2016;388:3036–3047. 27265346 10.1016/S0140-6736(16)30654-7

[2] Zunt JR, Kassebaum NJ, Blake N, et al. Global, regional, and national burden of meningitis, 1990–2016: a systematic analysis for the global burden of disease study 2016. Lancet Neurol. 2018;17:1061–1082. 30507391 10.1016/S1474-4422(18)30387-9 PMC6234314

[3] van de Beek D, Brouwer M, Hasbun R, et al. Community-acquired bacterial meningitis. Nat Rev Dis Primers. 2016;2:16074. 27808261 10.1038/nrdp.2016.74

[4] Coureuil M, Lecuyer H, Bourdoulous S, et al. A journey into the brain: insight into how bacterial pathogens cross blood-brain barriers. Nature Rev Microbiol. 2017;15:149–159. 28090076 10.1038/nrmicro.2016.178

[5] Join-Lambert O, Morand PC, Carbonnelle E, et al. Mechanisms of meningeal invasion by a bacterial extracellular pathogen, the example of Neisseria meningitidis. Prog Neurobiol. 2010;91:130–139. 20026234 10.1016/j.pneurobio.2009.12.004

[6] Dando SJ, Mackay-Sim A, Norton R, et al. Pathogens penetrating the central nervous system: infection pathways and the cellular and molecular mechanisms of invasion. Clin Microbiol Rev. 2014;27:691–726. 25278572 10.1128/CMR.00118-13 PMC4187632

[7] Kim KS. Mechanisms of microbial traversal of the blood-brain barrier. Nature Rev Microbiol. 2008;6:625–634. 18604221 10.1038/nrmicro1952 PMC5206914

[8] Coureuil M, Mikaty G, Miller F, et al. Meningococcal type IV pili recruit the polarity complex to cross the brain endothelium. Science. 2009;325:83–87. 19520910 10.1126/science.1173196 PMC3980637

[9] Nikulin J, Panzner U, Frosch M, et al. Intracellular survival and replication of Neisseria meningitidis in human brain microvascular endothelial cells. Int J Med Microbiol. 2006;296:553–558. 17010667 10.1016/j.ijmm.2006.06.006

[10] Kim BJ, Hancock BM, Bermudez A, et al. Bacterial induction of Snail1 contributes to blood-brain barrier disruption. J Clin Invest. 2015;125:2473–2483. 25961453 10.1172/JCI74159 PMC4497739

[11] Nizet V, Kim KS, Stins M, et al. Invasion of brain microvascular endothelial cells by group B streptococci. Infect Immun. 1997;65:5074–5081. 9393798 10.1128/iai.65.12.5074-5081.1997 PMC175731

[12] Cutting AS, Del Rosario Y, Mu R, et al. The role of autophagy during group B Streptococcus infection of blood-brain barrier endothelium. J Biol Chem. 2014;289:35711–35723. 25371213 10.1074/jbc.M114.588657 PMC4276841

[13] Krishnan S, Fernandez GE, Sacks DB, et al. IQGAP1 mediates the disruption of adherens junctions to promote Escherichia coli K1 invasion of brain endothelial cells. Cell Microbiol. 2012;14:1415–1433. 22519731 10.1111/j.1462-5822.2012.01805.x PMC3410974

[14] Huang SH, Wass C, Fu Q, et al. Escherichia coli invasion of brain microvascular endothelial cells in vitro and in vivo: molecular cloning and characterization of invasion gene ibe10. Infect Immun. 1995;63:4470–4475. 7591087 10.1128/iai.63.11.4470-4475.1995 PMC173636

[15] Gao X, Qian J, Zheng S, et al. Overcoming the blood-brain barrier for delivering drugs into the brain by using adenosine receptor nanoagonist. ACS Nano. 2014;8:3678–3689. 24673594 10.1021/nn5003375

[16] Carman AJ, Mills JH, Krenz A, et al. Adenosine receptor signaling modulates permeability of the blood-brain barrier. J Neurosci. 2011;31:13272–13280. 21917810 10.1523/JNEUROSCI.3337-11.2011 PMC3328085

[17] Bynoe MS, Viret C, Yan A, et al. Adenosine receptor signaling: a key to opening the blood-brain door. Fluids Barriers CNS. 2015;12:20. 26330053 10.1186/s12987-015-0017-7 PMC4557218

[18] Kim DG, Bynoe MS. A2A adenosine receptor regulates the human blood-brain barrier permeability. Mol Neurobiol. 2015;52:664–678. 25262373 10.1007/s12035-014-8879-2 PMC4439385

[19] Mills JH, Alabanza L, Weksler BB, et al. Human brain endothelial cells are responsive to adenosine receptor activation. Purinergic Signal. 2011;7:265–273. 21484089 10.1007/s11302-011-9222-2 PMC3146641

[20] Mills JH, Thompson LF, Mueller C, et al. CD73 is required for efficient entry of lymphocytes into the central nervous system during experimental autoimmune encephalomyelitis. Proc Natl Acad Sci U S A. 2008;105:9325–9330. 18591671 10.1073/pnas.0711175105 PMC2453691

[21] Segura M, Zheng H, de Greeff A, et al. Latest developments on Streptococcus suis: an emerging zoonotic pathogen: part 2. Future Microbiol. 2014;9:587–591. 24957086 10.2217/fmb.14.15

[22] Tang J, Wang C, Feng Y, et al. Streptococcal toxic shock syndrome caused by Streptococcus suis serotype 2. PLoS Med. 2006;3:e151. 16584289 10.1371/journal.pmed.0030151 PMC1434494

[23] Liu P, Pian Y, Li X, et al. Streptococcus suis adenosine synthase functions as an effector in evasion of PMN-mediated innate immunity. J Infect Dis. 2014;210:35–45. 24446521 10.1093/infdis/jiu050

[24] Thammavongsa V, Kern JW, Missiakas DM, et al. Staphylococcus aureus synthesizes adenosine to escape host immune responses. J Exp Med. 2009;206:2417–2427. 19808256 10.1084/jem.20090097 PMC2768845

[25] Firon A, Dinis M, Raynal B, et al. Extracellular nucleotide catabolism by the group B Streptococcus ectonucleotidase NudP increases bacterial survival in blood. J Biol Chem. 2014;289:5479–5489. 24429288 10.1074/jbc.M113.545632 PMC3937624

[26] Fan J, Zhang Y, Chuang-Smith ON, et al. Ecto-5ʹ-nucleotidase: a candidate virulence factor in Streptococcus sanguinis experimental endocarditis. PLoS One. 2012;7:e38059. 22685551 10.1371/journal.pone.0038059 PMC3369921

[27] Strazza M, Maubert ME, Pirrone V, et al. Co-culture model consisting of human brain microvascular endothelial and peripheral blood mononuclear cells. J Neurosci Methods. 2016;269:39–45. 27216631 10.1016/j.jneumeth.2016.05.016 PMC4925208

[28] Dominguez-Punaro MC, Segura M, Plante MM, et al. Streptococcus suis serotype 2, an important swine and human pathogen, induces strong systemic and cerebral inflammatory responses in a mouse model of infection. J Immunol. 2007;179:1842–1854. 17641051 10.4049/jimmunol.179.3.1842

[29] Kong D, Chen Z, Wang J, et al. Interaction of factor H-binding protein of Streptococcus suis with globotriaosylceramide promotes the development of meningitis. Virulence. 2017;8:1290–1302. 28402705 10.1080/21505594.2017.1317426 PMC5711355

[30] Chang YC, Wang Z, Flax LA, et al. Glycosaminoglycan binding facilitates entry of a bacterial pathogen into central nervous systems. PLoS Pathog. 2011;7:e1002082. 21731486 10.1371/journal.ppat.1002082 PMC3121876

[31] Chang YC, Olson J, Beasley FC, et al. Group B Streptococcus engages an inhibitory Siglec through sialic acid mimicry to blunt innate immune and inflammatory responses in vivo. PLoS Pathog. 2014;10:e1003846. 24391502 10.1371/journal.ppat.1003846 PMC3879367

[32] Doran KS, Liu GY, Nizet V. Group B streptococcal β-hemolysin/cytolysin activates neutrophil signaling pathways in brain endothelium and contributes to development of meningitis. J Clin Investig. 2003;112:736–744. 12952922 10.1172/JCI17335 PMC182187

[33] Doran KS, Engelson EJ, Khosravi A, et al. Blood-brain barrier invasion by group B Streptococcus depends upon proper cell-surface anchoring of lipoteichoic acid. J Clin Invest. 2005;115:2499–2507. 16138192 10.1172/JCI23829 PMC1193870

[34] Yang T, Gao X, Sandberg M, et al. Abrogation of adenosine A1 receptor signalling improves metabolic regulation in mice by modulating oxidative stress and inflammatory responses. Diabetologia. 2015;58:1610–1620. 25835725 10.1007/s00125-015-3570-3

[35] Gorska AM, Golembiowska K. The role of adenosine A1 and A2A receptors in the caffeine effect on MDMA-induced DA and 5-HT release in the mouse striatum. Neurotox Res. 2015;27:229–245. 25391902 10.1007/s12640-014-9501-0 PMC4353865

[36] Synnestvedt K, Furuta GT, Comerford KM, et al. Ecto-5ʹ-nucleotidase (CD73) regulation by hypoxia-inducible factor-1 mediates permeability changes in intestinal epithelia. J Clin Invest. 2002;110:993–1002. 12370277 10.1172/JCI15337 PMC151145

[37] Shechter R, Miller O, Yovel G, et al. Recruitment of beneficial M2 macrophages to injured spinal cord is orchestrated by remote brain choroid plexus. Immunity. 2013;38:555–569. 23477737 10.1016/j.immuni.2013.02.012 PMC4115271

[38] Ran FA, Hsu PD, Wright J, et al. Genome engineering using the CRISPR-Cas9 system. Nat Protoc. 2013;8:2281–2308. 24157548 10.1038/nprot.2013.143 PMC3969860

[39] Sanjana NE, Shalem O, Zhang F. Improved vectors and genome-wide libraries for CRISPR screening. Nat Methods. 2014;11:783–784. 25075903 10.1038/nmeth.3047 PMC4486245

[40] Mincham KT, Scott NM, Lauzon-Joset JF, et al. Transplacental immune modulation with a bacterial-derived agent protects against allergic airway inflammation. J Clin Invest. 2018;128:4856–4869. 30153109 10.1172/JCI122631 PMC6205372

[41] Sato H, Silveira L, Spagnolo P, et al. CC chemokine receptor 5 gene polymorphisms in beryllium disease. Eur Respir J. 2010;36:331–338. 20075058 10.1183/09031936.00107809 PMC3061572

[42] Fowler VG Jr., Das AF, Lipka-Diamond J, et al. Exebacase for patients with Staphylococcus aureus bloodstream infection and endocarditis. J Clin Invest. 2020;130:3750–3760. 10.1172/JCI136577 PMC7324170 32271718

[43] Shi Y, Zhang L, Pu H, et al. Rapid endothelial cytoskeletal reorganization enables early blood-brain barrier disruption and long-term ischaemic reperfusion brain injury. Nat Commun. 2016;7:10523. 26813496 10.1038/ncomms10523 PMC4737895

[44] Eigenmann DE, Xue G, Kim KS, et al. Comparative study of four immortalized human brain capillary endothelial cell lines, hCMEC/D3, hBMEC, TY10, and BB19, and optimization of culture conditions, for an in vitro blood-brain barrier model for drug permeability studies. Fluids Barriers CNS. 2013;10:33. 24262108 10.1186/2045-8118-10-33 PMC4176484

[45] Shi Y, Jiang X, Zhang L, et al. Endothelium-targeted overexpression of heat shock protein 27 ameliorates blood-brain barrier disruption after ischemic brain injury. Proc Natl Acad Sci U S A. 2017;114:E1243–E52. 28137866 10.1073/pnas.1621174114 PMC5320958

[46] Chen J, Luo Y, Hui H, et al. CD146 coordinates brain endothelial cell-pericyte communication for blood-brain barrier development. Proc Natl Acad Sci U S A. 2017;114:E7622–E31. 28827364 10.1073/pnas.1710848114 PMC5594696

[47] Charland N, Nizet V, Rubens CE, et al. Streptococcus suis serotype 2 interactions with human brain microvascular endothelial cells. Infect Immun. 2000;68:637–643. 10639427 10.1128/iai.68.2.637-643.2000 PMC97186

[48] Williams AE, Blakemore WF. Pathogenesis of meningitis caused by Streptococcus suis type 2. J Infect Dis. 1990;162:474–481. 2373874 10.1093/infdis/162.2.474

[49] Madsen LW, Svensmark B, Elvestad K, et al. Streptococcus suis serotype 2 infection in pigs: new diagnostic and pathogenetic aspects. J Comp Pathol. 2002;126:57–65. 11814322 10.1053/jcpa.2001.0522

[50] Sanford SE. Gross and histopathological findings in unusual lesions caused by Streptococcus suis in pigs. I. Cardiac lesions. Can J Vet Res. 1987;51:481–485. 3453268 PMC1255369

[51] Tenenbaum T, Papandreou T, Gellrich D, et al. Polar bacterial invasion and translocation of Streptococcus suis across the blood-cerebrospinal fluid barrier in vitro. Cell Microbiol. 2009;11:323–336. 19046337 10.1111/j.1462-5822.2008.01255.x

[52] Koshiba M, Kojima H, Huang S, et al. Memory of extracellular adenosine A2A purinergic receptor-mediated signaling in murine T cells. J Biol Chem. 1997;272:25881–25889. 9325320 10.1074/jbc.272.41.25881

[53] Cronstein BN, Sitkovsky M. Adenosine and adenosine receptors in the pathogenesis and treatment of rheumatic diseases. Nat Rev Rheumatol. 2017;13:41–51. 27829671 10.1038/nrrheum.2016.178 PMC5173391

[54] Argaw AT, Gurfein BT, Zhang Y, et al. VEGF-mediated disruption of endothelial CLN-5 promotes blood-brain barrier breakdown. Proc Natl Acad Sci U S A. 2009;106:1977–1982. 19174516 10.1073/pnas.0808698106 PMC2644149

[55] Feoktistov I, Goldstein AE, Ryzhov S, et al. Differential expression of adenosine receptors in human endothelial cells: role of A2B receptors in angiogenic factor regulation. Circ Res. 2002;90:531–538. 11909816 10.1161/01.res.0000012203.21416.14

[56] Leibovich SJ, Chen JF, Pinhal-Enfield G, et al. Synergistic up-regulation of vascular endothelial growth factor expression in murine macrophages by adenosine A(2A) receptor agonists and endotoxin. Am J Pathol. 2002;160:2231–2244. 12057925 10.1016/S0002-9440(10)61170-4 PMC1850844

[57] Ryzhov S, Novitskiy SV, Zaynagetdinov R, et al. Host A(2B) adenosine receptors promote carcinoma growth. Neoplasia. 2008;10:987–995. 18714400 10.1593/neo.08478 PMC2517644

[58] Caporarello N, Olivieri M, Cristaldi M, et al. Blood-brain barrier in a haemophilus influenzae type a in vitro infection: role of adenosine receptors A2A and A2B. Mol Neurobiol. 2018;55:5321–5336. 28921456 10.1007/s12035-017-0769-y

[59] Zagursky RJ, Ooi P, Jones KF, et al. Identification of a Haemophilus influenzae 5ʹ-nucleotidase protein: cloning of the nucA gene and immunogenicity and characterization of the NucA protein. Infect Immun. 2000;68:2525–2534. 10768940 10.1128/iai.68.5.2525-2534.2000 PMC97455

[60] Estrela AB, Turck P, Stutz E, et al. Release of periplasmic nucleotidase induced by human antimicrobial peptide in E. coli causes accumulation of the immunomodulator adenosine. PLoS One. 2015;10:e0138033. 26371472 10.1371/journal.pone.0138033 PMC4570785

