# Supplementary material for: Bacteria elevate extracellular adenosine to exploit host signaling for blood-brain barrier disruption
Source: Virulence. 2020 Aug 10;11(1):980–94. doi: 10.1080/21505594.2020.1797352 (PMC7549952; doi:10.1080/21505594.2020.1797352)
Supplement: Supplemental Material [file KVIR_A_1797352_SM7951.zip › Suppl caption.docx]

**SUPPLEMENTAL MATERIAL**

Supplemental methods for this article may be found at **Supplemental Methods.**

**Figure S1. Bacterial loads in the blood of mice during the early period of *S. suis* infection.**

(**a**) Six-week-old female C57BL/6 mice were infected i.p. with approximately 5×10^6^ CFU of *S. suis* WT strain (*n* = 8) or the *ssads*-deficient mutant Δ*ssads* (*n* = 8). (**b**) Six-week-old female C57BL/6 mice were infected i.v. with approximately 1×10^8^ CFU of *S. suis* WT strain (*n* = 8) or the *ssads*-deficient mutant Δ*ssads* (*n* = 8). Bacterial counts in the blood (CFU/mL blood) were monitored at the indicated time points over the first 24 h post-infection.

**Figure S2. The effect of *ssads* deficiency on bacterial growth rate of *S. suis* strains.**

(**a**-**b**) Growth of ***S. suis*** WT strain and Δ*ssads* mutant in THY medium when 1% subcultured from stationary phase. The growth was monitored by measuring the OD_600 nm_ (**a**) with a spectrophotometer, or determining viable counts (**b**) via plating serial dilutions of each culture at regular time points. (**c**) Growth curves for *S. suis* Δ*ssads* mutant as compared to wild type *S. suis* strain in the upper chamber medium for transwell model used in Figure 2. Bacteria counts were determined by plating serial dilutions on THB agar plates.

**Figure S3. The effect of *ssads* deficiency on *S. suis* adhesion and invasion of HCMEC/D3 cells.**

HCMEC/D3 cells were infected with *S. suis* at an MOI of 100 for 1 h. For adherence (**a**), the cells were washed 4 times, and cracked by 1-mL PBS containing 0.1% saponin. The bacteria in the suspension were quantified by serial dilution plating. For invasion (**b**), the cells were washed 4 times, and then treated with 1-mL EBM-2 media containing 100 mg of gentamicin and 5 mg of penicillin G for another 1 h. After washing 3 times, the cells were cracked by 1-mL PBS containing 0.1% saponin. The bacteria in the suspension were quantified by serial dilution plating.

**Figure S4. The effect of the NECA treatment on translocation of *S. suis* WT across HCMEC/D3 monolayers.**

After HCMEC/D3 cells formed confluent and tight monolayers on transwell filters, *S. suis* WT strain were added to the apical surfaces of monolayers at an MOI of 100 in the presence or absence of adenosine analog NECA (1 µM). (**a**) *S. suis* cells in the lateral chamber after 1 h were quantitated; (**b**) The permeability coefficient to Lucifer yellow (LY) of HCMEC/D3 monolayers was measured 1 h post-infection. D-mannitol (10 µM) was used as a positive control as it disrupts cell-cell junctions. Data are expressed as means and SEM. No significant difference (n.s.) where P > 0.05, 2-tailed Student’s *t* test. (**c**) The TEER in HCMEC/D3 monolayers infected with *S. suis* WT strain in the presence or absence of NECA (1 µM) was measured. D-mannitol (10 µM) was used as a positive control as it disrupts cell-cell junctions. Data are expressed as means and SEM. Statistical significances were assessed using two-way analysis of ANOVA. No significant difference (n.s.) of TEER alteration induced by the *S. suis* WT infection between exogenous application of NECA (1 µM) and vehicle.

**Figure S5. Genome engineering used in the study to generate *Adora1* knockout cells and *Adora1* knockout mice.**

(**a-c**) *Adora1* knockout cells were generated from HCMEC/D3 cells using CRISPR/Cas9 genome editing. Insertion of a purine base into the 43–44 base of exon 1 of *Adora1* using CRISPR/Cas9 genome editing in HCMEC/D3 cells gene caused a frameshift mutation and a premature stop codon (#) (**a**). Homozygous knockout cell lines were confirmed by sequencing of PCR fragments (**b**). Western blotting results demonstrating that the A1 AR was effectively depleted in *Adora1* knockout cells (**c**). (**d**) *Adora1* knockout mouse model (C57BL/6) generated by CRISPR/Cas-mediated genome engineering. Exon 2 was selected as target site, and a 1030-bp deletion was performed. The deletion was comfirmed by PCR and nucleic acid electrophoresis.

**Figure S6. Phylogenetic analysis of identified or predicted 5'-nucleotidase in bacterial species.**

Representative sequences from identified 5'-nucleotidase clusters with the enzyme activity were submitted to a BLAST analysis in order to recover other bacterial translation products harboring the adenosine synthase domain of these identified 5'-nucleotidase. The phylogenetic tree including the 22 identified or predicted 5'-nucleotidase sequences was constructed using MEGA software (7.0) under neighbor-joining method and p-distance model. The common or infrequent causative agents of meningitis were marked in red or violet, respectively.

**Figure S7. 5'-Nucleotidase activity assa**y **using different concentrations of metal cation.**

The 5'-nucleotidase activity of several bacterial species (1 × 10^8^ CFU/mL) was measured through the release of inorganic phosphate (Pi) from 50 µM AMP in the presence of metal cations (Ca^2+^ and/or Mg^2+^) over a period of 30 min. The tested bacteria included *S. suis*, *L. monocytogenes*, GBS, *S. aureus*, *S. epidermidis*, and *S. pneumoniae*.

**Figure S8. The translocation of *S. pneumoniae* across monolayers of HCMEC/D3 unedited cells or HCMEC/D3 A1 AR-KO cells.**

(**a**) After HCMEC/D3 unedited cells formed confluent and tight monolayers on transwell filters, ***S. pneumoniae*** strain were added to the apical surfaces of monolayers at an MOI of 100 in the presence or absence of APCP (500 µM), a known 5'-nucleotidase inhibitor. ***S. pneumoniae*** cells in the lateral chamber after 1 h were quantitated; (b) The effect of APCP (500 µM) on translocation of ***S. pneumoniae*** across monolayers of HCMEC/D3 A1 AR-KO cells. ***S. pneumoniae*** cells in the lateral chamber after 1 h were quantitated. Data are expressed as means and SEM. No significant difference (n.s.) where P > 0.05, 2-tailed Student’s *t* test.

**Table S1: Gram-positive bacteria harboring genes encoding a 5'-nucleotidase.**

**Table S2: Gram-negative bacteria harboring genes encoding a 5'-nucleotidase.**
