# Supplementary material for: Bacteria elevate extracellular adenosine to exploit host signaling for blood-brain barrier disruption
Source: Virulence. 2020 Aug 10;11(1):980–94. doi: 10.1080/21505594.2020.1797352 (PMC7549952; doi:10.1080/21505594.2020.1797352)
Supplement: Supplemental Material [file KVIR_A_1797352_SM7951.zip › Supplementary Methods.docx]

**Supplementary Methods**

**Mouse model of hematogenous meningitis**

The challenge protocol for *S. suis* infection was adapted from a murine model of *S. suis* meningitis using an intraperitoneal infection route ^1, 2^. Briefly, *S. suis* cells (in early of stationary phase) were washed once and diluted in THY medium prior to infection. Six-week-old female C57BL/6 mice (Vital River) or C57BL/6 A1 AR-KO mice were injected intraperitoneally (i.p.) with 1 mL of the bacterial suspensions (5×10^6^ colony-forming units (CFU)/mL) or vehicle alone (sterile THY media). Bacterial counts in venous blood were examined at 5 h or 16 h post-infection to ensure mice developed similar degrees of bacteremia. Seventy-two hours post-infection, mice were euthanized and samples of venous blood and brain were collected aseptically. Bacterial counts in blood and tissue homogenates were determined by plating serial dilutions on THB agar plates. Brain bacterial counts were corrected for blood contamination using the blood concentration and a conservative estimate of the mouse cerebral blood volume ^3-6^. In some experiments, brain samples or brain hemispheres were collected for histopathologic analysis. For GBS infection, a well-defined and widely-used mouse model of hematogenous GBS meningitis was used as described previously ^3-7^. Briefly, 6-week-old male CD1 mice (Vital River) were injected via tail vein with 10^8^ CFU of GBS. Bacterial CFUs in venous blood were examined at 16 h and 72 h post-infection and brain samples were collected aseptically at 72 h post-infection. CCPA (0.37 mg/kg BW) or vehicle was administered intravenously (i.v.) at 2 h post-infection. DPCPX (1 mg/kg BW) or vehicle was injected i.p. concomitantly with bacterial cells as described in previous studies ^8, 9^. APCP (20 mg/kg BW) or vehicle was injected i.p. 2 h prior to infection as described in previous studies ^10, 11^. The A1 AR-KO mice were obtained by using the CRISPR/Cas9 technique from Cyagen Biosciences. The targeting strategy is described at <https://www.cyagen.com/cn/zh-cn/sperm-bank/11539>.

**Knockout using CRISPR/Cas9 genome editing**

*Adora1* knockout (KO) cells were generated from HCMEC/D3 cells using CRISPR/Cas9 genome editing as described ^12, 13^. Briefly, a sgRNA was constructed targeting exon 1 of human *Adora1* and cloned into the lentiCRISPRv2 puro plasmid, a gift from Brett Stringer (Addgene plasmid # 98290). Cells were transiently transfected with the corresponding plasmids using Lipofectamine 2000 (Invitrogen). Preliminary screening was performed in the presence of 12 µg/mL puromycin due to high expression of P-glycoprotein in brain endothelial cell cultures ^14^. Single cell clones were isolated by limiting dilution and the purity of KO cells was confirmed by sequencing of PCR (polymerase chain reaction) fragments and by immunoblotting.

**Isolation of primary murine primary brain microvascular endothelial cells**

Primary murine brain microvascular endothelial cells (pMBMECs) were isolated and purified using a well-established and previously-described method ^3^. Briefly, the cerebral cortices from 6-week-old female C57BL/6 mice were used as the source of microvessels. The first digestion was conducted using a mixture of collagenase type 2 (270 U/mL; Worthington) and DNAse I (20 U/mL; TransGen Biotech) in Dulbecco’s modified Eagle medium (DMEM) for 1.5 h (hours) at 37°C with shaking at 180 rpm. DMEM containing 20% bovine serum albumin (BSA; Amresco) was used to remove myelin. The second digestion was conducted using a mixture of collagenase type 2 (140 U/mL), dispase (1 U/mL; Worthington) and DNAse I (20 U/mL) in DMEM for 1 h at 37°C with shaking at 180 rpm. The remaining glial cells and red blood cells were removed using 33% continuous Percoll gradient centrifugation as described ^15^. The pMBMEC cells were originally plated on 60-mm TC-treated dishes (Corning) coated with 2 µg/cm^2^ fibronectin. DMEM supplemented with 20% FBS, 1 ng/mL basic fibroblast growth factor, 100 µg/mL heparin (Sigma-Aldrich; only added in early culture), and 1.4 µM hydrocortisone (TOCRIS) was used to culture pMBMECs. To purify the endothelium, puromycin (8 µg/mL) (Sigma-Aldrich) was added to the media for the first 72 h. The pMBMEC cells were passaged at 80% confluence once prior to experiments.

**Translocation of *S. suis* across endothelial monolayers.**

Briefly, endothelial cells were seeded (approximately 1×10^4^ cells) on the apical side of collagen-coated 3.0-μm pore Millicell inserts (Merck Millipore) in 24-well plates (Corning) and grown for 4 to 5 days until intact monolayers were formed. The cells were serum starved (0.25% FBS) for 12–18 h before the experiments. Confluent endothelial monolayers (approximately 1×10^5^ cells) were incubated with bacteria (MOI = 100; 1×10^7^ CFU bacteria in 0.2 mL media) in the apical chamber for 1 h. FBS (50%) was also added into the upper chamber medium to simulate the metal cation content of blood. After infection, medium from the lower chamber was collected and sampled to quantitate the number of viable bacteria that had crossed the monolayer by plating the lower chamber medium on THB agar plates. Relative bacterial transmigration was corrected for differences between the amounts of bacteria in the upper chamber. For Lucifer yellow (LY) permeability, LY (50 μM) was added to upper chamber. After incubation, the LY fluorescence of the media in lower chamber was measured by MD SpectraMax i3 system (Molecular Devices) at absorption/emission of 428/536 nm. The permeability coefficient to LY of HCMEC/D3 monolayers was determined. TEER values were measured using the Millicell® ERS-2 Electrical Resistance System (Merck Millipore) according to the manufacturer’s instructions.

**Determination of the intracellular cAMP concentration**

Briefly, confluent endothelial monolayers in collagen-coated 6-well plates were incubated with *S. suis* WT strain or the Δ*ssads* mutant at an MOI of 100 for 1 h. The cells were washed twice with ice-cold PBS and lysates were assayed immediately according to the manufacturer’s protocol, using cAMP parameter assay (R&D Systems).

**Western blotting**

Briefly, endothelial cells after various experimental treatments were washed once with ice-cold PBS (pH 7.2) and lysed on ice with lysis buffer (25 mM HEPES, pH 7.5, containing 150 mM NaCl, 1% NP-40, 10 mM MgCl_2_, 1 mM EDTA (ethylenediamine tetraacetic acid), 2% glycerol, and 2.5 mM Na_3_VO_4_) supplemented with phosphatase cocktail (Roche) and complete mini protease inhibitor (Roche). Cell lysates were harvested with a cell scraper, snap frozen in liquid nitrogen, and stored at -70°C. All frozen lysates were thawed in a room temperature water bath and centrifuged at 13,000 g at 4°C for 10 min. The supernatant was mixed with 6 × protein loading buffer (Transgen biotech) and boiled for 5 min (minutes) at 100°C. The protein samples were separated by SDS-PAGE (sodium dodecylsulfate-polyacrylamide gel electrophoresis) and transferred to nitrocellulose or PVDF (polyvinylidene difluoride) membrane. The membranes were blocked with 5% BSA in TBS (triethanolamine-buffered saline) for 1 h at room temperature and then incubated with primary antibody in TBS containing 0.1% Tween-20 (TBST) and 3% BSA overnight at 4°C. Primary antibodies against the following proteins were used: mouse anti-GAPDH (1:5,000; Invitrogen MA5-15738), rabbit anti-A1 AR (1:200; Alomone AAR-006). Subsequently, membranes were washed with TBST and incubated with IRDye 800CW-conjugated goat anti-rabbit IgG secondary antibody (1:5,000; Li-Cor 926-32211) or IRDye® 680RD-conjugated goat anti-mouse IgG secondary antibody (1:5,000; Li-Cor 926-68070) in TBST containing 3% BSA for 1 h at room temperature. Membranes were washed with TBST and visualized using an Odyssey Infrared Imaging System (Li-Cor Biosciences). Relative band quantification was assessed using LI-COR Image Studio Lite Version 3.1.

**Immunocytochemistry**

The pMBMEC cells were grown on fibronectin-coated Millicell EZ SLIDEs 4-well glass slides (Merck Millipore). After starvation, confluent endothelial monolayers (approximately 2.5×10^5^ cells) were incubated with bacteria at an MOI of 10 for 5 h, followed by gently rising once in warm PBS (phosphate-buffered saline). The cells were fixed with warm 3.7% paraformaldehyde for 12 min, permeabilized with 0.2% Triton X-100 for 5 min, and blocked with 5% BSA in PBS for 1 h at room temperature. The cells were then incubated with rabbit anti-ZO-1 antibody (1:50; Invitrogen 61-7300) overnight at 4°C. After rinsing in PBS, cells were incubated with goat anti-rabbit IgG antibody conjugated to Alexa Fluor® 488 (1:1,000; CST 4412) at room temperature for 1 h. F-actin was stained with rhodamine phalloidin (75 nM; Cytoskeleton). The slides were mounted using SlowFade Diamond Antifade Mountant with DAPI (4',6-diamidino-2-phenylindole; Invitrogen) and visualized with a confocal laser scanning microscope (Olympus FV1000).

**Bioinformatics and phylogenetic analysis of Ssads orthologs in other bacteria**

Representative sequences from Ssads or other identified 5'-nucleotidase clusters with the enzyme activity ^16-20^ were submitted to a Basic Local Alignment Search Tool (BLAST) analysis in order to recover other bacterial translation products harboring the adenosine synthase domain of these identified 5'-nucleotidase. The BLAST analysis was performed using sequences obtained from GenBank database. Evolutionary analyses were conducted in MEGA 7.0 and phylogenetic tree was generated using the Neighbor-Joining method ^21^ under p-distance model ^22^. The common or infrequent causative agents of meningitis were marked in red or violet, respectively.

**5'-Nucleotidase activity assay**

The 5'-nucleotidase activity exhibited by whole cells of several bacterial species was measured by detection of inorganic phosphate released from AMP hydrolysis using a QuantiChrom Phosphate Assay Kit DIPI-500 (Bioassay systems). The results were expressed as μM of Pi converted from 50 μM AMP by 10^8^ bacterial cells within 30 min at the desired concentration of divalent metal ions.

**Measurement of bacterial growth rate**

*S. suis* strains were streaked onto Columbia sheep blood agar plates and incubated at 37°C in a 5% CO_2_-enriched atmosphere overnight then subcultured in THY growth medium to stationary phase. Columbia sheep blood agar plates containing 5μg/mL chloramphenicol were used for *S. suis* Δ*ssads* culture. The two strains were subcultured to fresh antibiotic-free THY medium at a ratio of 1%. The growth was monitored by measuring the OD_600_ nm with a spectrophotometer and determining viable counts via plating serial dilutions of each culture at the indicated time points. To test the growth rate of *S. suis* strains in the upper chamber of transwell model, fresh EBM-2 medium with 50% FBS were inoculated with the two strains at an initial concentration 5×10^6^ CFU/mL. Viable counts were determined by plating serial dilutions of each culture at the indicated time points.

**Supplementary References**

1. Dominguez-Punaro MC, Segura M, Plante MM, Lacouture S, Rivest S, Gottschalk M. Streptococcus suis serotype 2, an important swine and human pathogen, induces strong systemic and cerebral inflammatory responses in a mouse model of infection. J Immunol 2007; 179:1842-54.

2. Kong D, Chen Z, Wang J, Lv Q, Jiang H, Zheng Y, et al. Interaction of factor H-binding protein of Streptococcus suis with globotriaosylceramide promotes the development of meningitis. Virulence 2017; 8:1290-302.

3. Chang YC, Wang Z, Flax LA, Xu D, Esko JD, Nizet V, et al. Glycosaminoglycan binding facilitates entry of a bacterial pathogen into central nervous systems. PLoS Pathog 2011; 7:e1002082.

4. Chang YC, Olson J, Beasley FC, Tung C, Zhang J, Crocker PR, et al. Group B Streptococcus engages an inhibitory Siglec through sialic acid mimicry to blunt innate immune and inflammatory responses in vivo. PLoS Pathog 2014; 10:e1003846.

5. Doran KS, Liu GY, Nizet V. Group B streptococcal β-hemolysin/cytolysin activates neutrophil signaling pathways in brain endothelium and contributes to development of meningitis. Journal of Clinical Investigation 2003; 112:736-44.

6. Doran KS, Engelson EJ, Khosravi A, Maisey HC, Fedtke I, Equils O, et al. Blood-brain barrier invasion by group B Streptococcus depends upon proper cell-surface anchoring of lipoteichoic acid. J Clin Invest 2005; 115:2499-507.

7. Kim BJ, Hancock BM, Bermudez A, Del Cid N, Reyes E, van Sorge NM, et al. Bacterial induction of Snail1 contributes to blood-brain barrier disruption. J Clin Invest 2015; 125:2473-83.

8. Yang T, Gao X, Sandberg M, Zollbrecht C, Zhang XM, Hezel M, et al. Abrogation of adenosine A1 receptor signalling improves metabolic regulation in mice by modulating oxidative stress and inflammatory responses. Diabetologia 2015; 58:1610-20.

9. Gorska AM, Golembiowska K. The role of adenosine A1 and A2A receptors in the caffeine effect on MDMA-induced DA and 5-HT release in the mouse striatum. Neurotox Res 2015; 27:229-45.

10. Synnestvedt K, Furuta GT, Comerford KM, Louis N, Karhausen J, Eltzschig HK, et al. Ecto-5'-nucleotidase (CD73) regulation by hypoxia-inducible factor-1 mediates permeability changes in intestinal epithelia. J Clin Invest 2002; 110:993-1002.

11. Shechter R, Miller O, Yovel G, Rosenzweig N, London A, Ruckh J, et al. Recruitment of beneficial M2 macrophages to injured spinal cord is orchestrated by remote brain choroid plexus. Immunity 2013; 38:555-69.

12. Ran FA, Hsu PD, Wright J, Agarwala V, Scott DA, Zhang F. Genome engineering using the CRISPR-Cas9 system. Nature protocols 2013; 8:2281-308.

13. Sanjana NE, Shalem O, Zhang F. Improved vectors and genome-wide libraries for CRISPR screening. Nature methods 2014; 11:783-4.

14. Perriere N, Demeuse P, Garcia E, Regina A, Debray M, Andreux JP, et al. Puromycin-based purification of rat brain capillary endothelial cell cultures. Effect on the expression of blood-brain barrier-specific properties. J Neurochem 2005; 93:279-89.

15. Ruck T, Bittner S, Epping L, Herrmann AM, Meuth SG. Isolation of primary murine brain microvascular endothelial cells. Journal of visualized experiments : JoVE 2014:e52204.

16. Thammavongsa V, Kern JW, Missiakas DM, Schneewind O. Staphylococcus aureus synthesizes adenosine to escape host immune responses. J Exp Med 2009; 206:2417-27.

17. Firon A, Dinis M, Raynal B, Poyart C, Trieu-Cuot P, Kaminski PA. Extracellular nucleotide catabolism by the Group B Streptococcus ectonucleotidase NudP increases bacterial survival in blood. The Journal of biological chemistry 2014; 289:5479-89.

18. Zagursky RJ, Ooi P, Jones KF, Fiske MJ, Smith RP, Green BA. Identification of a Haemophilus influenzae 5'-nucleotidase protein: cloning of the nucA gene and immunogenicity and characterization of the NucA protein. Infect Immun 2000; 68:2525-34.

19. Fan J, Zhang Y, Chuang-Smith ON, Frank KL, Guenther BD, Kern M, et al. Ecto-5'-nucleotidase: a candidate virulence factor in Streptococcus sanguinis experimental endocarditis. PLoS One 2012; 7:e38059.

20. Estrela AB, Turck P, Stutz E, Abraham WR. Release of Periplasmic Nucleotidase Induced by Human Antimicrobial Peptide in E. coli Causes Accumulation of the Immunomodulator Adenosine. PLoS One 2015; 10:e0138033.

21. Saitou N, Nei M. The neighbor-joining method: a new method for reconstructing phylogenetic trees. Mol Biol Evol 1987; 4:406-25.

22. Nei M, Kumar S. Molecular evolution and phylogenetics. Oxford ; New York: Oxford University Press, 2000.
