# Supplementary material for: Bacteria elevate extracellular adenosine to exploit host signaling for blood-brain barrier disruption
Source: Virulence. 2020 Aug 10;11(1):980–94. doi: 10.1080/21505594.2020.1797352 (PMC7549952; doi:10.1080/21505594.2020.1797352)
Supplement: Supplemental Material [file KVIR_A_1797352_SM7951.zip › Table S1.docx]

**Table S1: Gram-positive bacteria harboring genes encoding a 5'-nucleotidase.**

| Organism | Function | Acession no.^a^ |
| --- | --- | --- |
| *Bacillus anthracis* | 2',3'-cyclic-nucleotide 2'-phosphodiesterase | Q6HTQ7 |
| *Bacillus cereus* | 5'-Nucleotidase domain protein | EEL51488.1 |
| *Clostridium perfringens* | 5'-nucleotidase family protein | ABG87133.1 |
| *Enterococcus faecalis* | 5'-nucleotidase family protein | Q839U0 |
| *Listeria monocytogenes* | bifunctional metallophosphatase/5'-nucleotidase | WP_010989350.1 |
| *Staphylococcus aureu* | possible 5'-nucleotidase | ABX28069.1 |
| *Staphylococcus epidermidis* | 5' nucleotidase family protein | Q5HQE0 |
| *Streptococcus agalactiae* | bifunctional metallophosphatase/5'-nucleotidase | ATZ91655.1 |
| *Streptococcus suis* | 5'-nucleotidase | WP_012775133.1 |
| *Streptococcus mutans* | Putative 5'-nucleotidase | Q8CVC5 |
| *Streptococcus gordonii* | bifunctional metallophosphatase/5'-nucleotidase | WP_012000643.1 |
| *Streptococcus pyogenes* | putative surface-anchored 5'-nucleotidase | A2RF30 |
| *Streptococcus sanguinis* | 5'-nucleotidase | YP_001035187.1 |

^a^ Available from GenBank.
