# Supplementary material for: Bacteria elevate extracellular adenosine to exploit host signaling for blood-brain barrier disruption
Source: Virulence. 2020 Aug 10;11(1):980–94. doi: 10.1080/21505594.2020.1797352 (PMC7549952; doi:10.1080/21505594.2020.1797352)
Supplement: Supplemental Material [file KVIR_A_1797352_SM7951.zip › Table S2.docx]

**Table S2: Gram-negative bacteria harboring genes encoding a 5'-nucleotidase.**

| Organism | Function | Acession no. |
| --- | --- | --- |
| *Escherichia coli* | 5'-nucleotidase | KQI97463.1 |
| *Haemophilus influenzae* | 5'-nucleotidase NucA | AAD33949.1 |
| *Klebsiella pneumoniae* | 5'-nucleotidase | SAV81274.1 |
| *Legionella pneumophila* | 5'-nucleotidase | CZR22874.1 |
| *Pasteurella multocida* | 5'-nucleotidase | KWW10247.1 |
| *Salmonella typhimurium* | 5'-nucleotidase | AMM01929.1 |
| *Shigella dysenteriae* Sd197 | 2',3'-cyclic-nucleotide 2'-phosphodiesterase | ABB64270.1 |
| *Shigella dysenteriae* | bifunctional UDP-sugar hydrolase/5'-nucleotidase | WP_011379033.1 |
| *Vibrio cholerae* | 5'-nucleotidase | KQA83734.1 |

^a^ Available from GenBank.
