# Supplementary figures and images for: Bacteria elevate extracellular adenosine to exploit host signaling for blood-brain barrier disruption
Source: Virulence. 2020 Aug 10;11(1):980–94. doi: 10.1080/21505594.2020.1797352 (PMC7549952; doi:10.1080/21505594.2020.1797352)

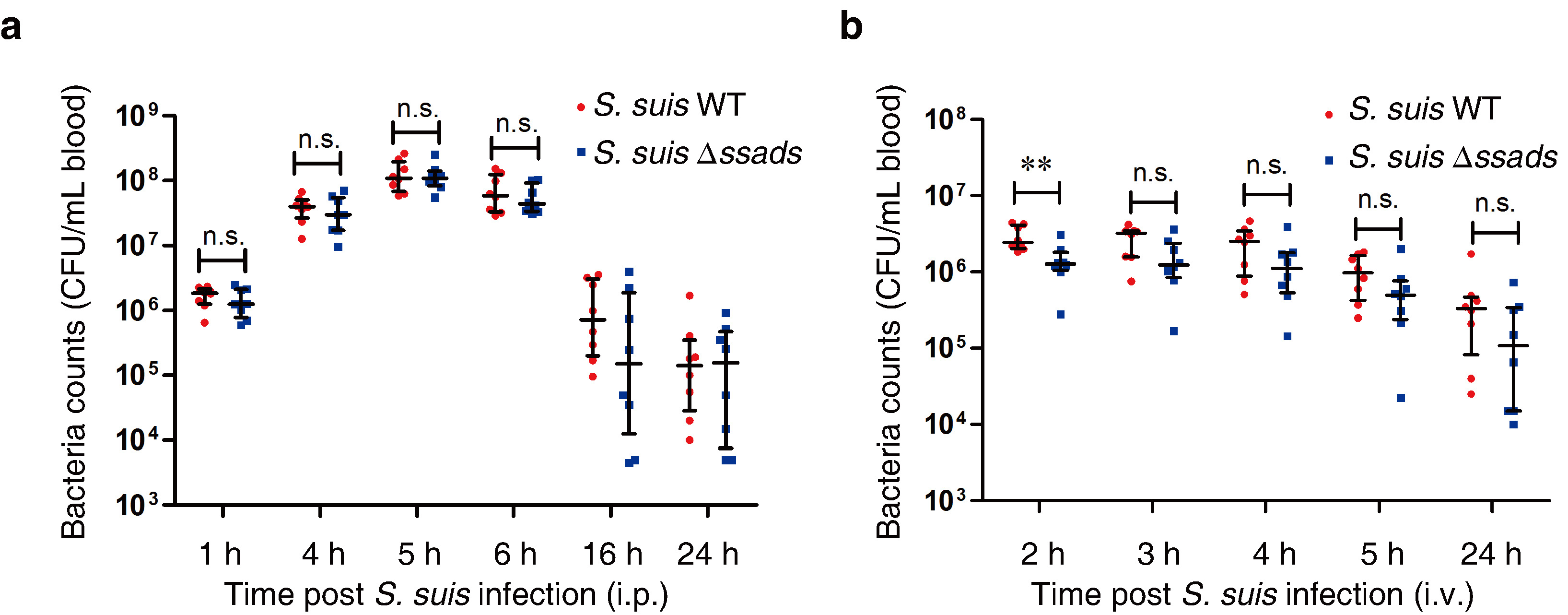

Supplement: Supplemental Material [file KVIR_A_1797352_SM7951.zip › Figure S1.jpg]

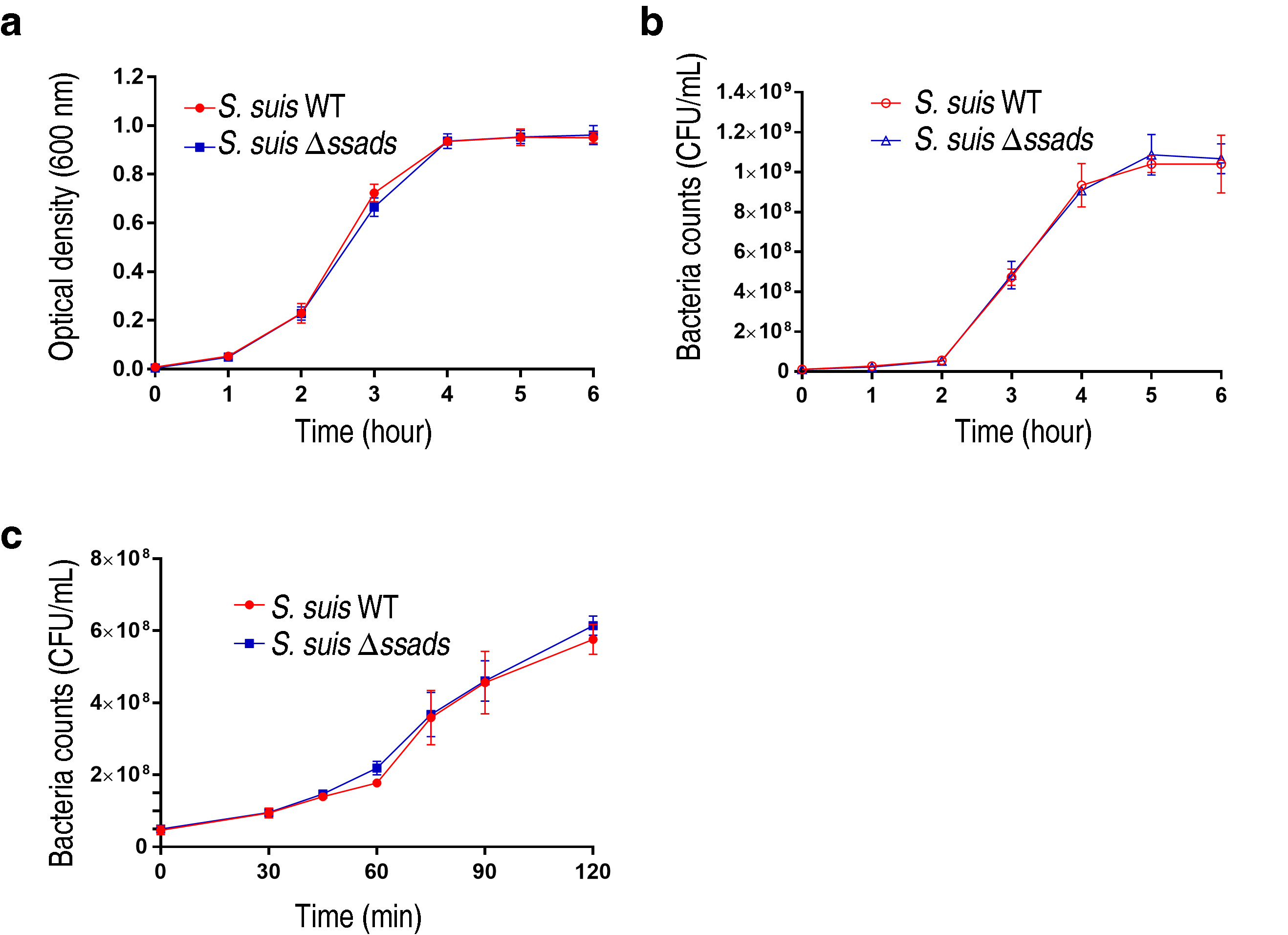

Supplement: Supplemental Material [file KVIR_A_1797352_SM7951.zip › Figure S2.jpg]

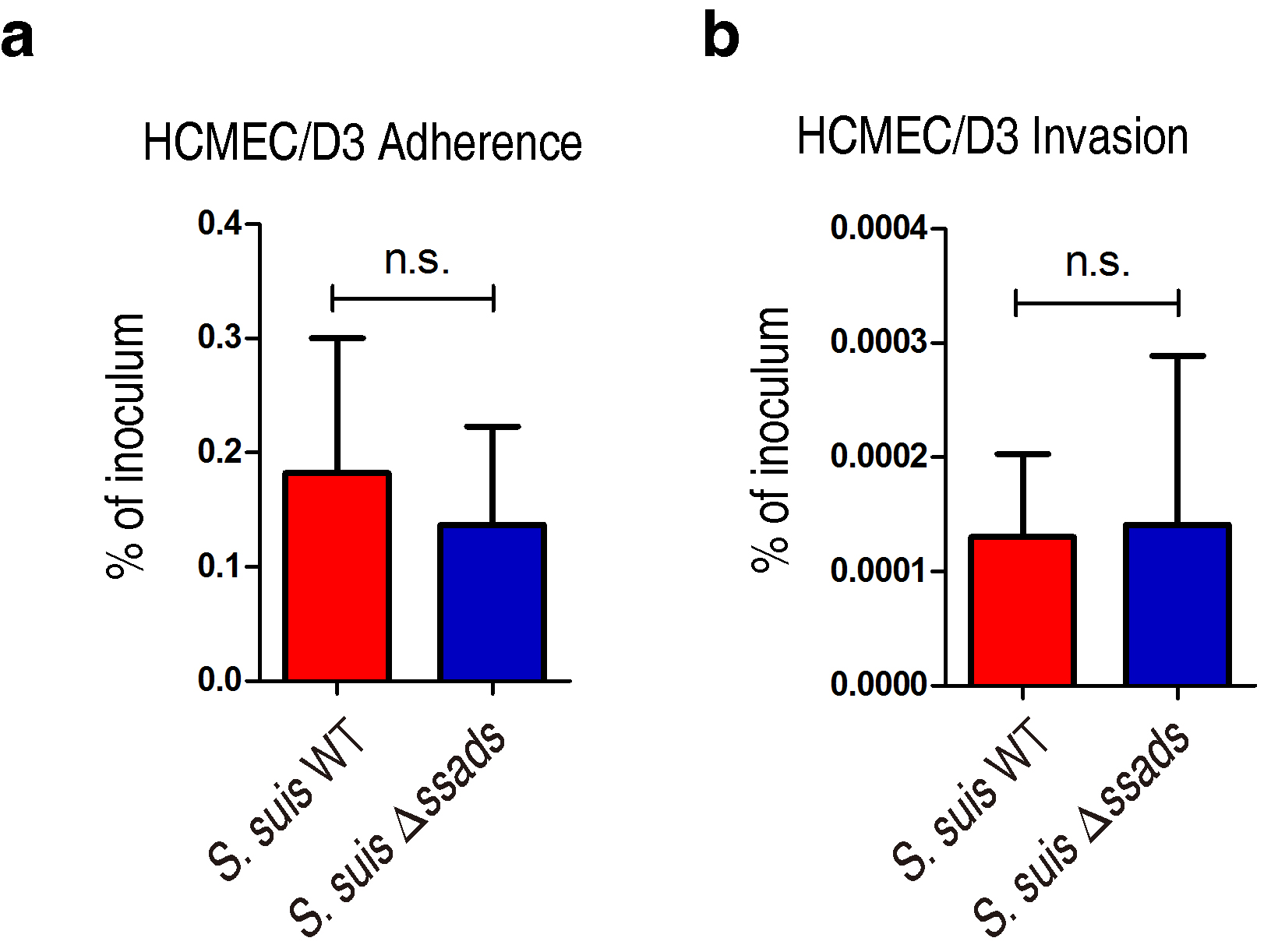

Supplement: Supplemental Material [file KVIR_A_1797352_SM7951.zip › Figure S3.jpg]

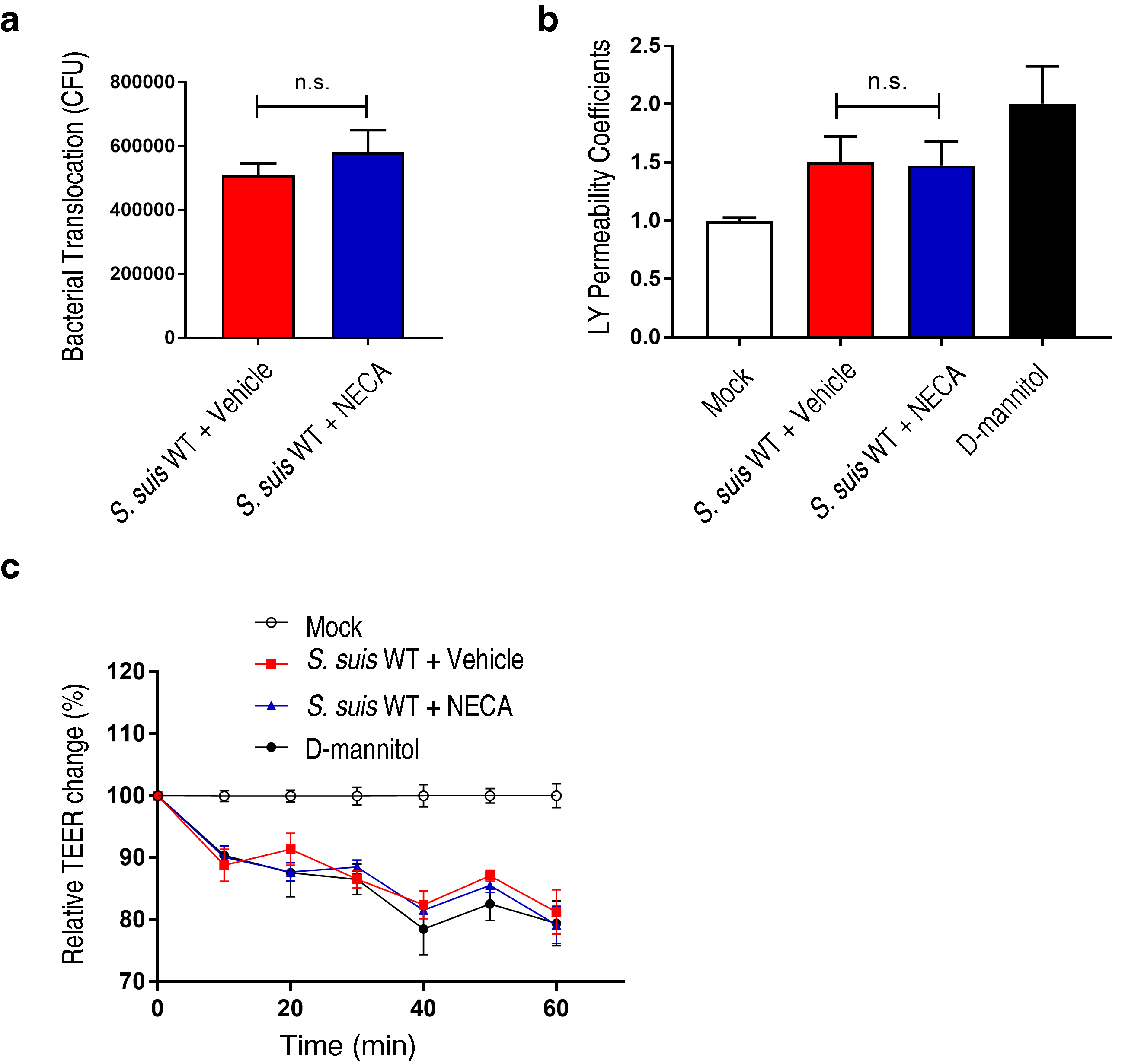

Supplement: Supplemental Material [file KVIR_A_1797352_SM7951.zip › Figure S4.jpg]

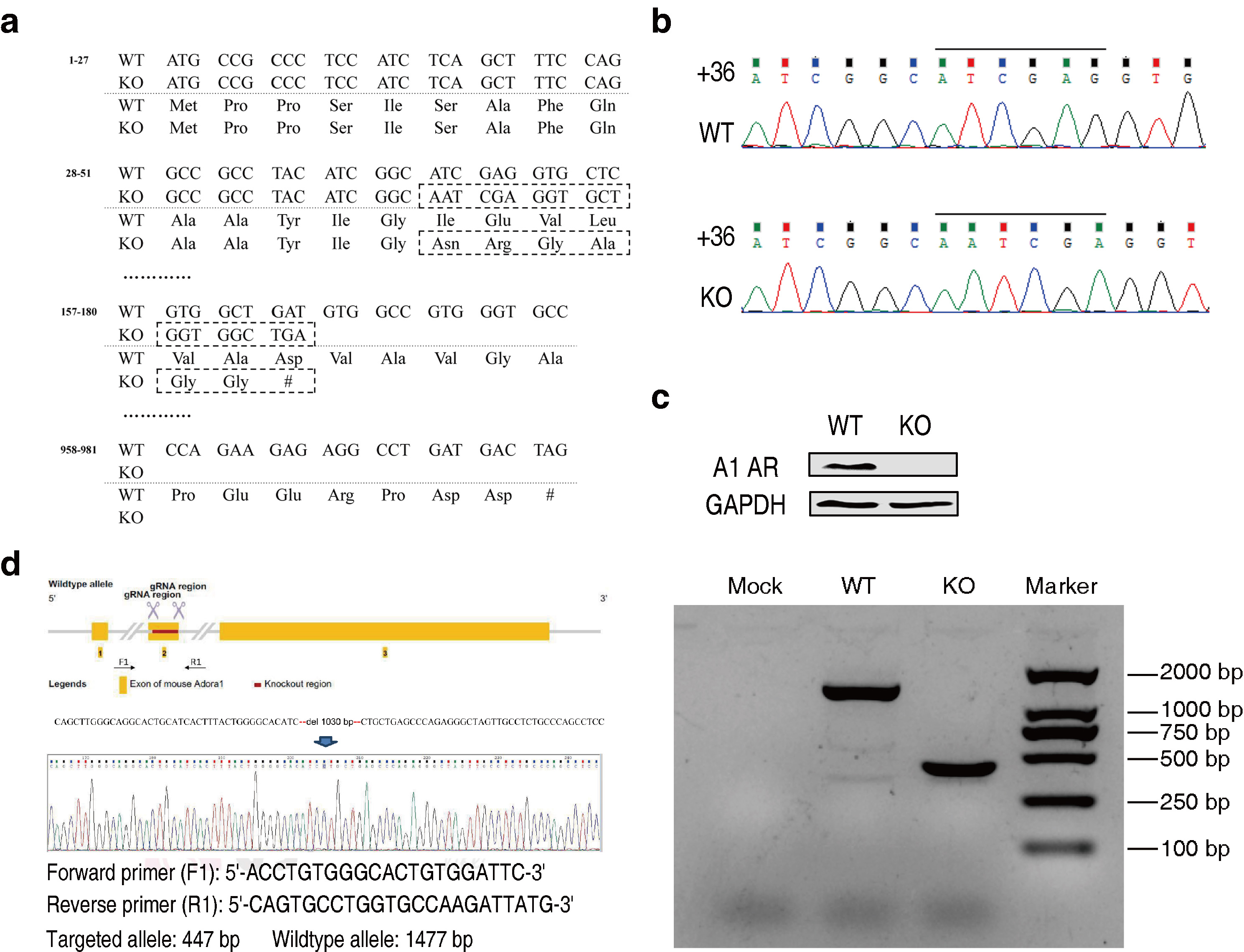

Supplement: Supplemental Material [file KVIR_A_1797352_SM7951.zip › Figure S5.jpg]

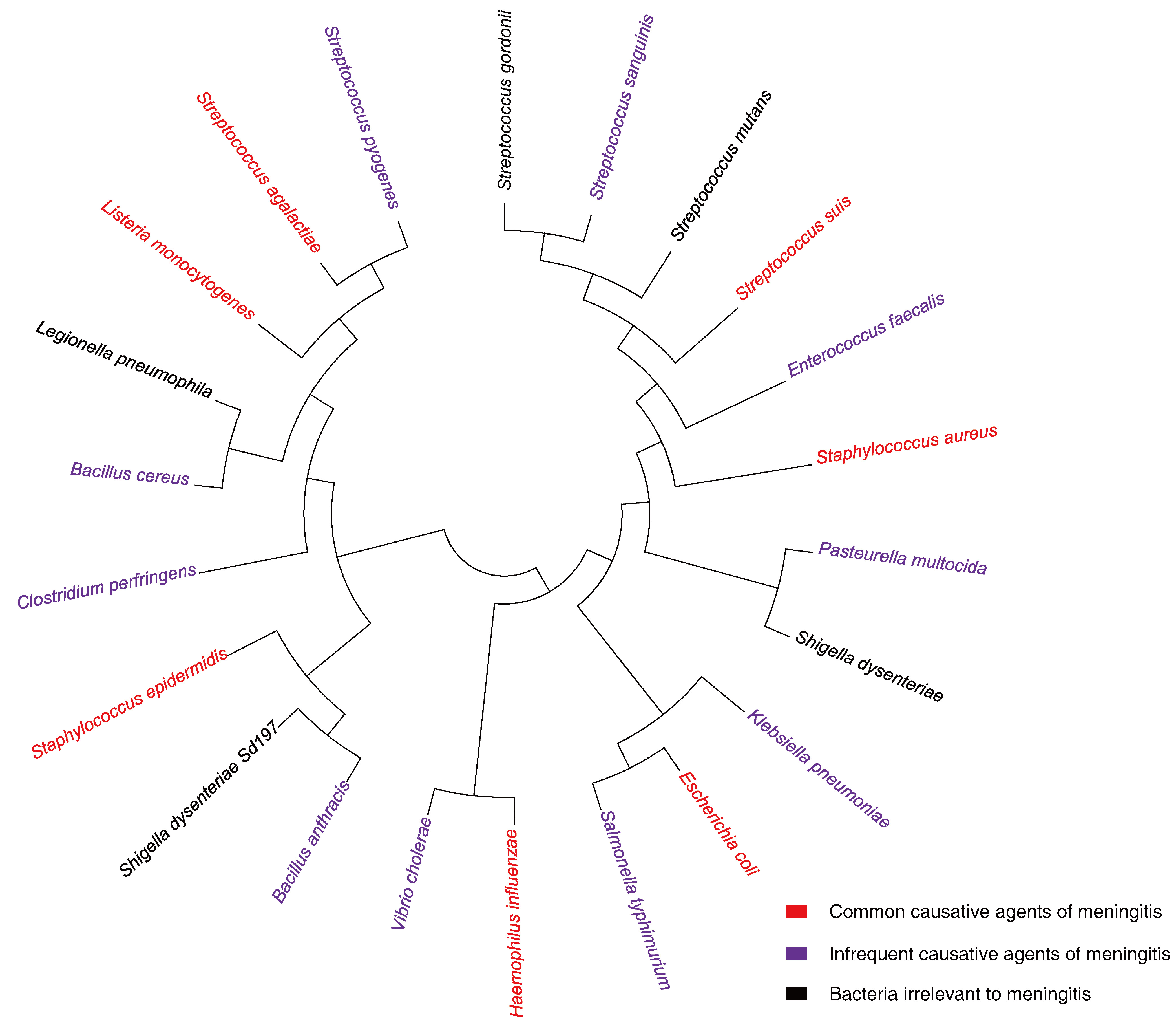

Supplement: Supplemental Material [file KVIR_A_1797352_SM7951.zip › Figure S6.jpg]

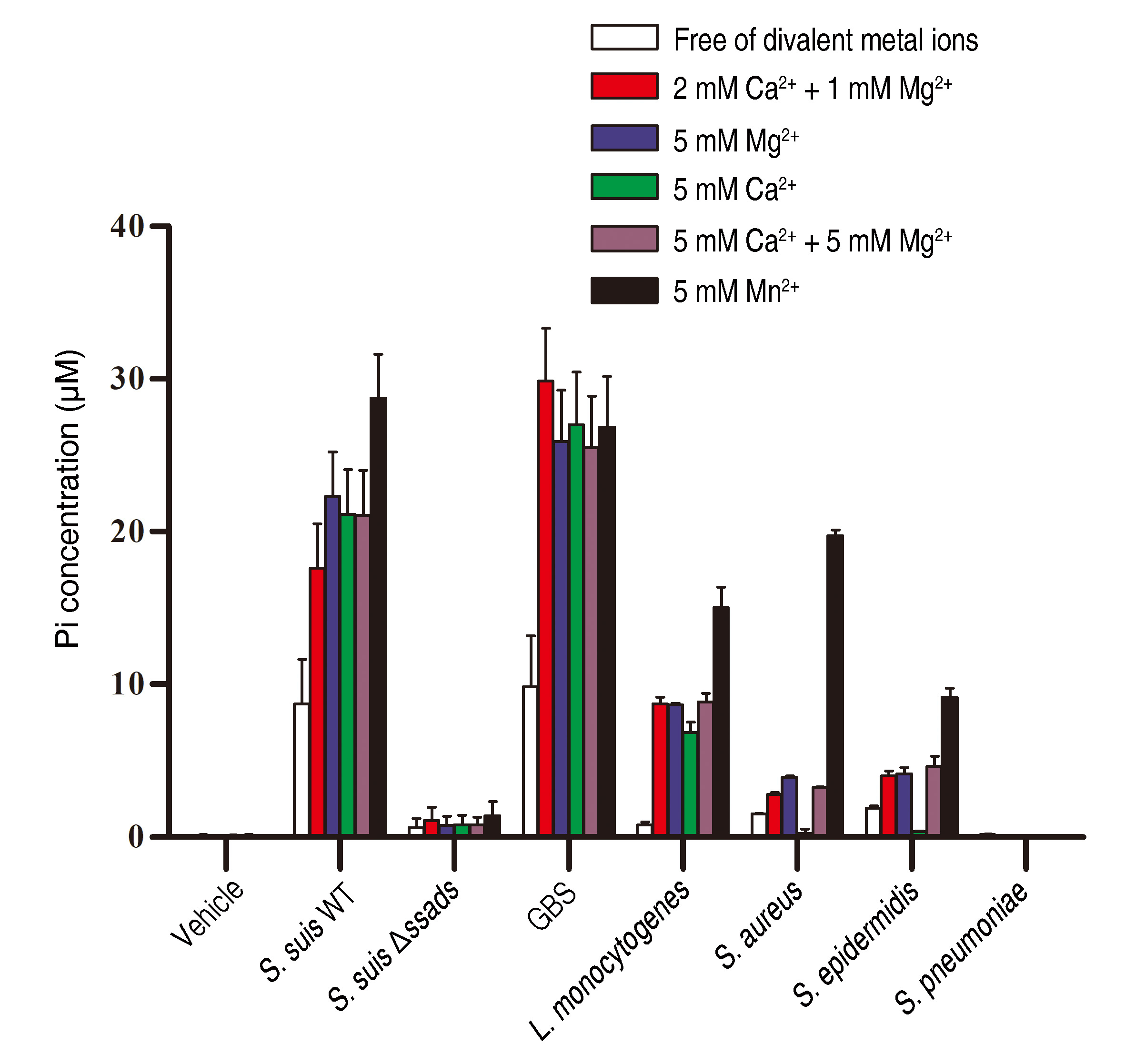

Supplement: Supplemental Material [file KVIR_A_1797352_SM7951.zip › Figure S7.jpg]

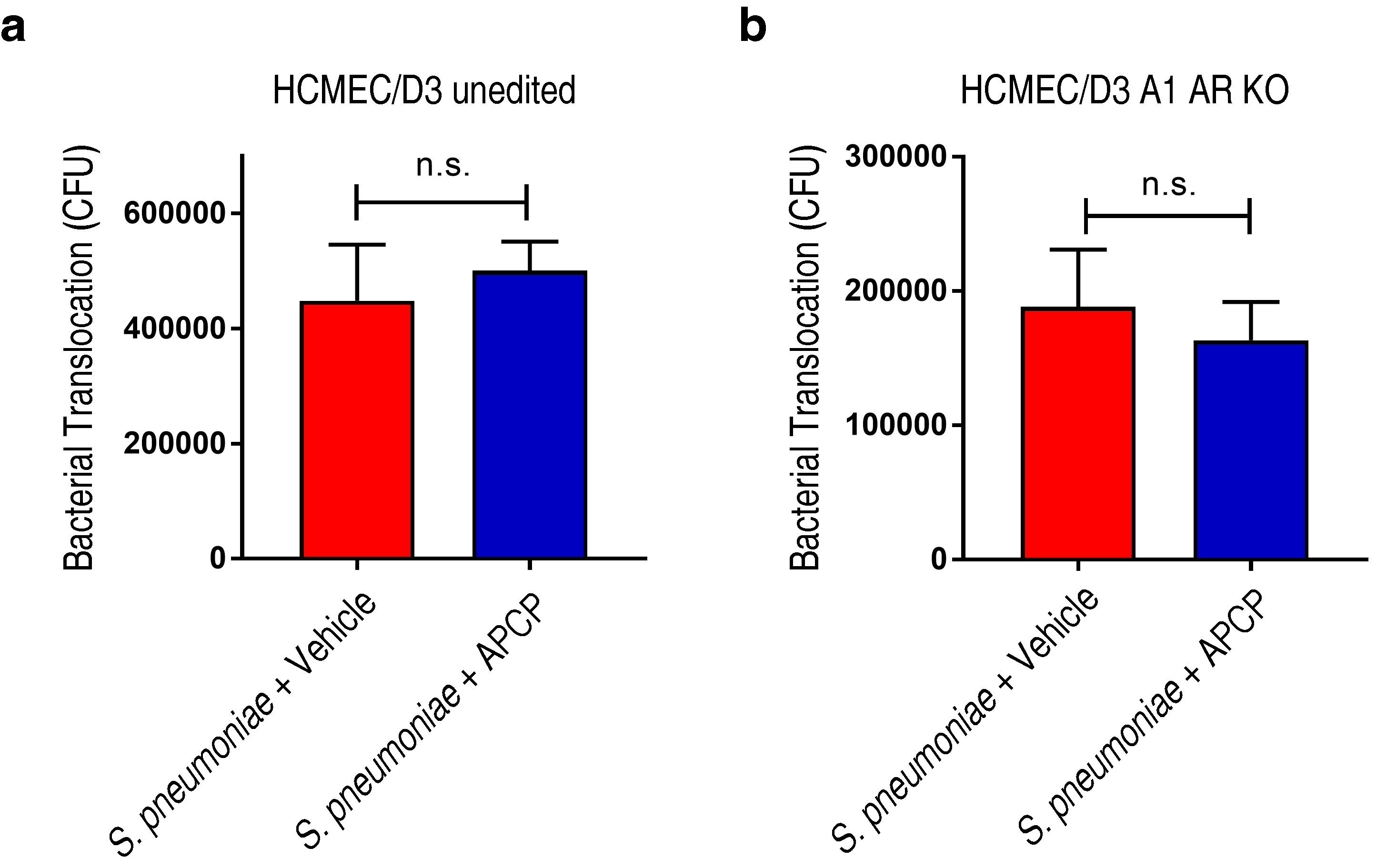

Supplement: Supplemental Material [file KVIR_A_1797352_SM7951.zip › Figure S8.jpg]
